# Genome Evolution in Bacteria Isolated from Million-Year-Old Subseafloor Sediment

**DOI:** 10.1128/mBio.01150-21

**Published:** 2021-08-17

**Authors:** William D. Orsi, Tobias Magritsch, Sergio Vargas, Ömer K. Coskun, Aurele Vuillemin, Sebastian Höhna, Gert Wörheide, Steven D’Hondt, B. Jesse Shapiro, Paul Carini

**Affiliations:** a Department of Earth and Environmental Sciences, Paleontology and Geobiology, Ludwig-Maximilians-Universität München, Munich, Germany; b GeoBio-CenterLMU, Ludwig-Maximilians-Universität München, Munich, Germany; c SNSB-Bayerische Staatssammlung für Paläontologie und Geologie, Munich, Germany; d Graduate School of Oceanography, University of Rhode Island, Narragansett, Rhode Island, USA; e Department of Biological Sciences, University of Montreal, Montreal, Quebec, Canada; f Department of Microbiology and Immunology, McGill University, Montreal, Quebec, Canada; g McGill Genome Centre, Montreal, Quebec, Canada; h Department of Environmental Science, University of Arizona, Tucson, Arizona, USA; i BIO5 Institute, University of Arizona, Tucson, Arizona, USA; j School of Plant Sciences, University of Arizona, Tucson, Arizona, USA; k School of Animal and Comparative Biomedical Science, University of Arizona, Tucson, Arizona, USA; University of California, Irvine

**Keywords:** genome evolution, deep biosphere, genomics, microbial evolution, phylogenomics

## Abstract

Beneath the seafloor, microbial life subsists in isolation from the surface world under persistent energy limitation. The nature and extent of genomic evolution in subseafloor microbes have been unknown. Here, we show that the genomes of *Thalassospira* bacterial populations cultured from million-year-old subseafloor sediments evolve in clonal populations by point mutation, with a relatively low rate of homologous recombination and elevated numbers of pseudogenes. Ratios of nonsynonymous to synonymous substitutions correlate with the accumulation of pseudogenes, consistent with a role for genetic drift in the subseafloor strains but not in type strains of *Thalassospira* isolated from the surface world. Consistent with this, pangenome analysis reveals that the subseafloor bacterial genomes have a significantly lower number of singleton genes than the type strains, indicating a reduction in recent gene acquisitions. Numerous insertion-deletion events and pseudogenes were present in a flagellar operon of the subseafloor bacteria, indicating that motility is nonessential in these million-year-old subseafloor sediments. This genomic evolution in subseafloor clonal populations coincided with a phenotypic difference: all subseafloor isolates have a lower rate of growth under laboratory conditions than the Thalassospira xiamenensis type strain. Our findings demonstrate that the long-term physical isolation of *Thalassospira*, in the absence of recombination, has resulted in clonal populations whereby reduced access to novel genetic material from neighbors has resulted in the fixation of new mutations that accumulate in genomes over millions of years.

## INTRODUCTION

The subseafloor biome contains a large fraction of all prokaryotic cells on Earth, totaling ca. 10^29^ cells (1). This biome subsists over geological timescales under persistent energy limitation (2–7). Whether evolution and ecological differentiation occur in microbial populations below the seafloor has remained controversial. It is generally agreed that extreme energy limitation restricts metabolic activity and growth (2–7), which are necessary for new mutations to propagate through populations to foster ecological differentiation and speciation (8). However, there has been very little direct examination of this issue at the low metabolic rates and long timescales characteristic of subseafloor life (9). Experimental evidence exists for bacterial evolution under energy limitation on laboratory timescales (10, 11), but a recent metagenomic analysis showed that energy limitation and reduced growth restricted the spread of new mutations through microbial communities over 5,000 years in the upper 2 m of anoxic sediment from Aarhus Bay (Denmark) (12). Moreover, the spread of new mutations is also limited in terrestrial deep subsurface bacterial populations in continental aquifers that appear to be surviving over geological timescales in evolutionary stasis (13). There have been no direct studies of mutation, homologous recombination, and evolution in microbial communities of the deeper and older sediment that dominates the subseafloor.

Newly acquired mutations of functional significance can sweep through bacterial populations in surface-world habitats and influence ecological differentiation (14), in large part due to their enormous effective population sizes (*N_e_*) (15). However, it is unclear whether such sweeps occur in ancient subseafloor sediment given the comparably low subseafloor bacterial biomass turnover rates that are estimated to be on thousand-year timescales (16) and the comparatively small *N_e_* for many populations (12). The metabolic rates of microbes persisting in deep-sea abyssal clay are among the lowest observed in the subseafloor biosphere such that these sediments are often oxic through the entire sediment column to the underlying oceanic crust, and the microbes live near the low-energy limit of life (2, 4). Here, we used the genomes of bacteria isolated from million-year-old subseafloor abyssal clay sediments to investigate the nature of genome evolution in subseafloor bacteria that persist under physical isolation from the surface world and each other over long timescales.

## RESULTS AND DISCUSSION

We cored a 15-m sedimentary sequence of oxygenated abyssal clay at a water depth of 6,000 m in the North Atlantic, where the average sedimentation rate is an estimated 1 m per million years (17). Thus, the deepest sediment sampled was deposited ca. 15 million years ago (mya). Abyssal clay is characterized by very low permeability and an extremely small pore diameter (18), which physically isolates the subseafloor microbial communities from the surface and the microbes within it from each other (see “Physical sediment properties” in Materials and Methods).

10.1128/mBio.01150-21.1 FIG S1 Summary of genome properties of *Thalassospira* strains used in this study. Points are derived from the analysis of existing genome sequences (for “type” strains) and new high-quality draft genomes sequenced as part of this study. Box plots illustrate interquartile ranges ± 1.5× the interquartile range. The horizontal line in each box plot is the median. NCBI genome accession numbers for the type strain genomes of *Thalassospira* used are as follows: NZ_JAATJD010000001.1 for *T. tepidiphila*, NZ_JPWA00000000.1 for *T. xianhensis*, NZ_ATWN01000001.1 for *T. lucentensis*, NZ_FTON01000032.1 for *T. xiamenensis*, NZ_AMRN01000001.1 for *T. profundimaris*, NZ_FNTU01000002.1 for *T. permensis*, NZ_JFKB00000000.1 for *T. alkalitolerans*, NZ_CP031555.1 for *T. indica*, NZ_CP024199.1 for *T. marina*, NZ_PGTS00000000.1 for *T. povalilytica*, NZ_NXGX00000000.1 for *T. lohafexi*, NZ_JFKA00000000.1 for *T. mesophila*, and NZ_JRJE00000000.1 for *T. australica*. Download FIG S1, PDF file, 0.1 MB. Copyright © 2021 Orsi et al. 2021 Orsi et al. https://creativecommons.org/licenses/by/4.0/ This content is distributed under the terms of the Creative Commons Attribution 4.0 International license.

**FIG 1:**
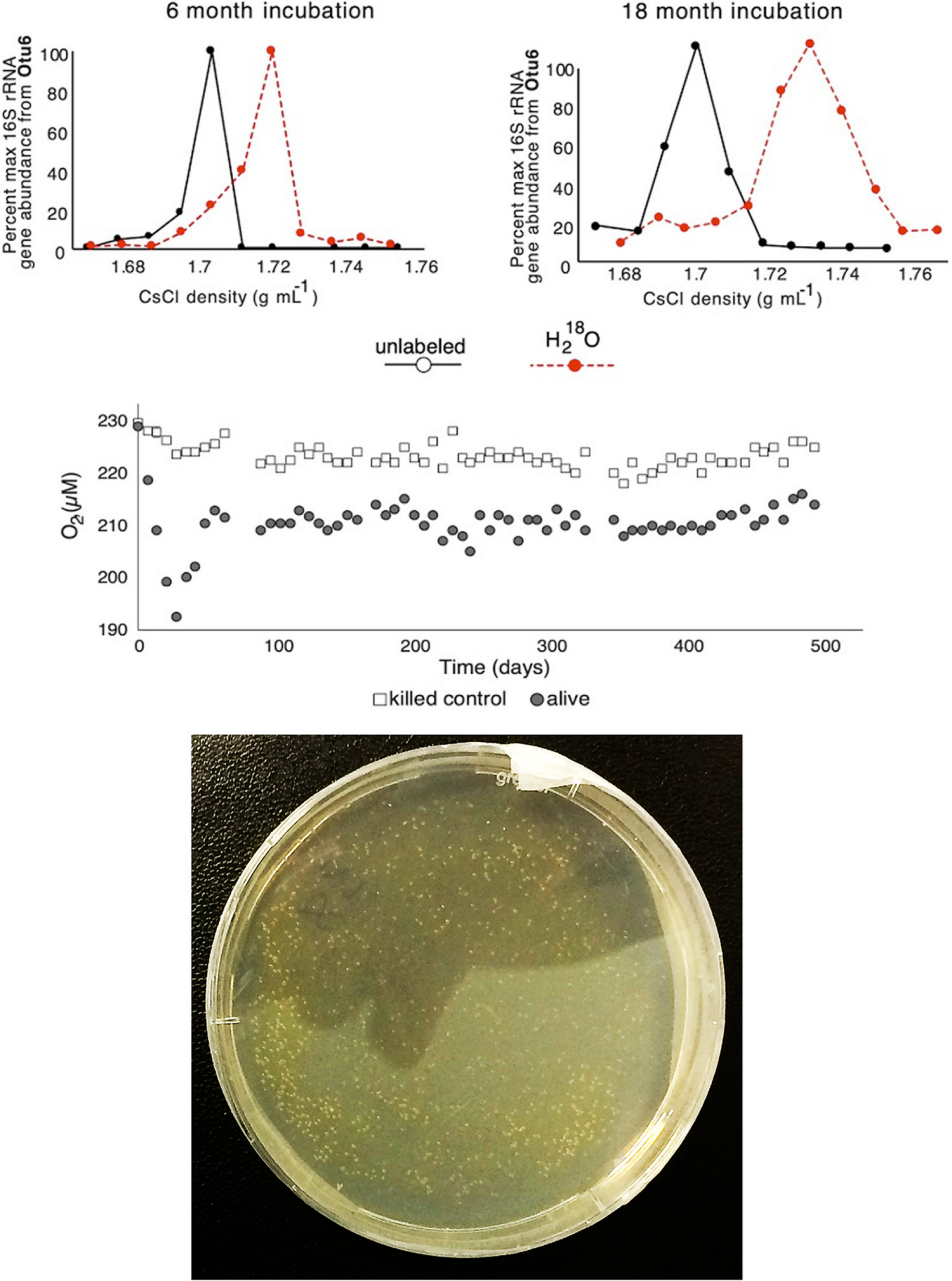
(Top) ^18^O labeling of 16S rRNA genes from *Thalassospira* OTU_6 after 6 and 18 months of incubation with ^18^O-labeled water from the 3-mbsf sediment (17). OTU_6 was one of >30 OTUs that were identified as ^18^O labeled and actively growing in the incubations (17). (Middle) Oxygen concentration over time in the 18-month slurry from the 3-mbsf incubation (filled circles) and slurries containing labeled water and autoclaved sediment (killed control). The O_2_ consumption rate was 0.04 μmol O_2_ liter^−1^ day^−1^. (Bottom) Cultivation of colony-forming bacteria on solid medium after the 18-month incubation of sediment slurries in sterile ^18^O-labeled artificial seawater. No bacterial colonies formed on petri dishes that were inoculated with the killed-control slurries. A total of 21 colonies were picked for genome sequencing, all of which were affiliated with the genus *Thalassospira*.

**FIG 2:**
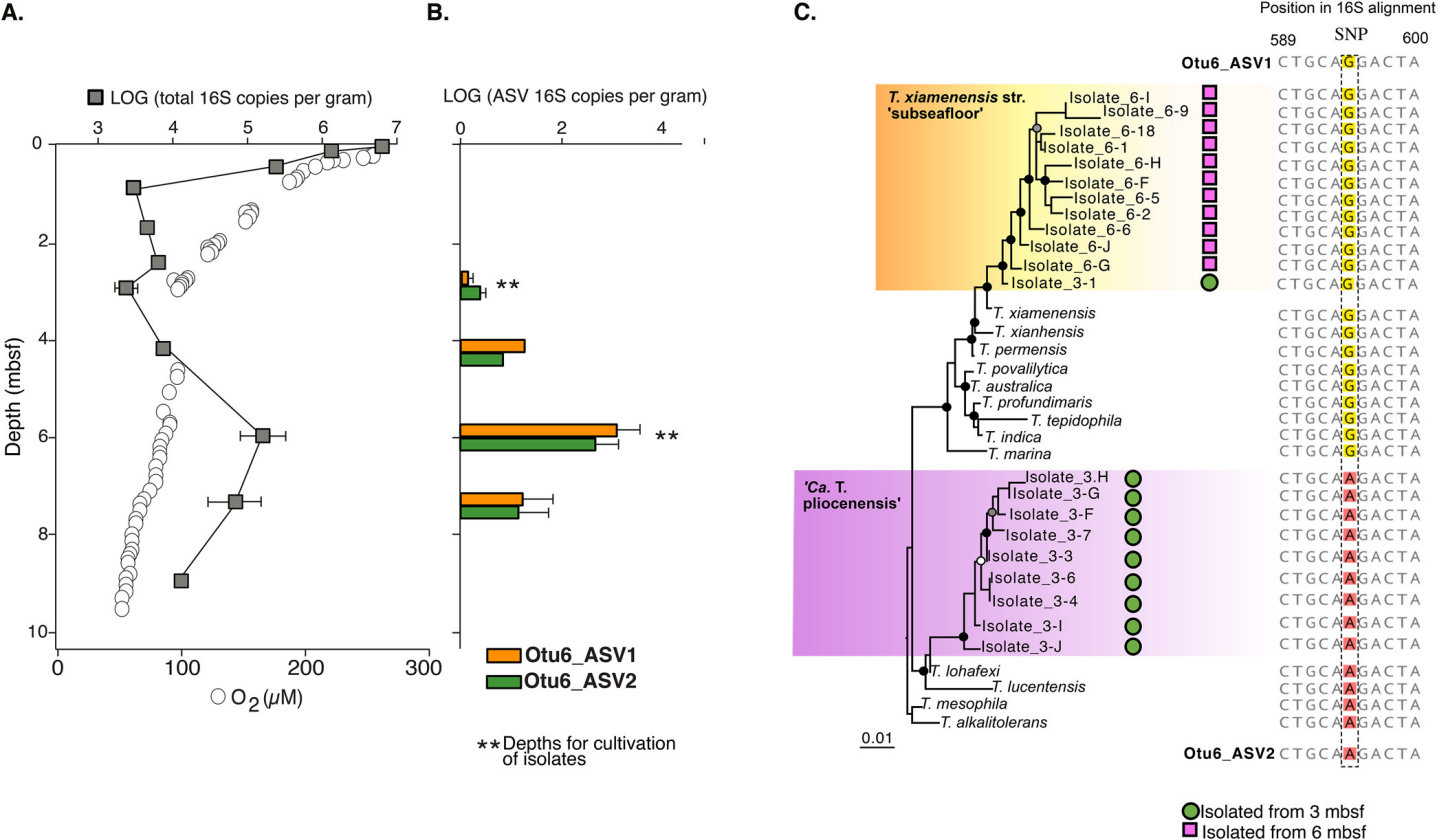
Isolated subsurface *Thalassospira* strains are most abundant at 3 to 7 mbsf and distinct from related type strains isolated from overlying water and sediments. (A) Vertical profile of total 16S rRNA gene concentrations determined by qPCR and oxygen concentrations. 16S rRNA gene concentration points are the average abundances from three technical qPCR replicates, with ranges shown with error bars. (B) qPCR-normalized average concentrations of the *Thalassospira*-affiliated OTU_6 ASVs. Error bars are ranges from three technical qPCR replicates. Asterisks mark the depths for the long-term ^18^O-water incubation experiments, enrichments, and cultivation. (C) Maximum likelihood (PhyML) phylogenetic analysis of the Sanger-sequenced, full-length *Thalassospira* 16S rRNA genes (alignment length, 1,554 nucleotides). The presence of the bifurcating SNPs is displayed, which is conserved between type strains, subseafloor strains, and the OTU_6 ASVs. Black, gray, and white dots at the nodes represent >90%, >70%, and >50% bootstrap support, respectively.

We isolated colony-forming bacteria on petri dishes following an 18-month incubation of sediment and sterile ^18^O-labeled artificial seawater from 3 and 6 m below the seafloor (mbsf) (Fig. 1 and 2). Because the mean sedimentation rate is 1 m million years^−1^, the ages of the sediment horizons from which these bacteria were enriched and isolated are estimated to be 3 million and 6 million years old, respectively. The full-length 16S rRNA gene sequences from the isolates had the closest similarity (98.5 to 99.5% sequence identity) to the alphaproteobacteria Thalassospira xiamenensis and Thalassospira lohafexi previously isolated from marine surface sediment (19, 20) and oligotrophic seawater (21) (these previously isolated microbes and their related cultured relatives are referred to as “type strains” here). The relatively slow drawdown of O_2_ with increasing depth at this site is due to the oxidation of organic matter by aerobic microbes (Fig. 2A).

Several lines of evidence indicate the *Thalassospira* isolated from the 3- and 6-mbsf sediment enrichments are endemic to the subseafloor clay and are not a contaminant from the water column or other sources. First, the V4 hypervariable regions of the 16S rRNA gene sequences from the sediment slurry-enriched *Thalassospira* cultures share >99% sequence identity with an operational taxonomic unit (OTU) previously identified from the *in situ* community determined from the frozen samples (“OTU_6”) (Fig. 2B and C). Second, OTU_6 became ^18^O labeled during the 18-month enrichment incubation (atomic ^18^O enrichment of OTU_6 DNA, 59%) in the presence of sterile ^18^O-labeled seawater (Fig. 1A), indicating that OTU_6 was growing in the incubation (22), with a mean estimated doubling time ± standard deviation (SD) of 36 ± 1.5 days. OTU_6 was one of >30 OTUs that were identified as ^18^O labeled and actively growing in the incubations (17), and it was the only ^18^O-labeled taxon from the enrichments that was detected as culturable on solid medium (Fig. 1). Third, a single nucleotide polymorphism (SNP) bifurcates OTU_6 into two classes of amplicon sequence variants (ASVs) (Fig. 2C). Both ASV classes were found in subsurface sediments (Fig. 2B) and in the 16S rRNA genes of the subseafloor isolates (Fig. 2C). Fourth, the *in situ* concentrations of both *Thalassospira* ASV classes have the highest abundances (ca. 1,000 16S rRNA gene copies g^−1^ sediment) below the seafloor between 4 and 6 mbsf, and both *Thalassospira* ASV classes were detected in the 3- and 6-mbsf sediments (Fig. 2B). These multiple lines of evidence show that the *Thalassospira* strains isolated from the 3- and 6-mbsf sediment enrichments are derived from the same distinct 16S rRNA gene ASV classes present within the *in situ* communities. The long-term physical isolation of these isolates in the subseafloor (see “Physical sediment properties” in Materials and Methods), subsisting under uninterrupted energy limitation within the ancient sediment, provides an opportunity to investigate how the relative effects of recombination, nucleotide substitution, and gene decay have shaped the genomes of the cultivated subsurface *Thalassospira* strains since their burial in the deep-sea clay millions of years ago.

### Genome statistics.

We sequenced the genomes of 21 *Thalassospira* isolates (10 from 3-mbsf and 11 from 6-mbsf sediments) using a hybrid assembly approach consisting of long-read Nanopore sequencing corrected and polished via short-read Illumina technologies at >100× coverage. The mean genome completeness of these hybrid assemblies is estimated to be 99.7% ± 0.3% (mean ± SD), with most being 100% complete and representing the complete chromosome (Table S1). The mean length of the new *Thalassospira* genomes is 4.71 ± 0.08 Mbp (mean ± SD), with 4,567 ± 107 (mean ± SD) protein-encoding genes, and they are assembled to an average of 12 ± 2 (mean ± SD) contigs (Fig. S1 and Table S1). The genome size and number of protein-encoding genes are similar to those observed within the existing *Thalassospira* type strains isolated from the surface world (Fig. S1).

10.1128/mBio.01150-21.6 TABLE S1 Genome summary statistics. Download Table S1, XLSX file, 0.01 MB. Copyright © 2021 Orsi et al. 2021 Orsi et al. https://creativecommons.org/licenses/by/4.0/ This content is distributed under the terms of the Creative Commons Attribution 4.0 International license.

### Core genome phylogenomic analysis.

**FIG 3:**
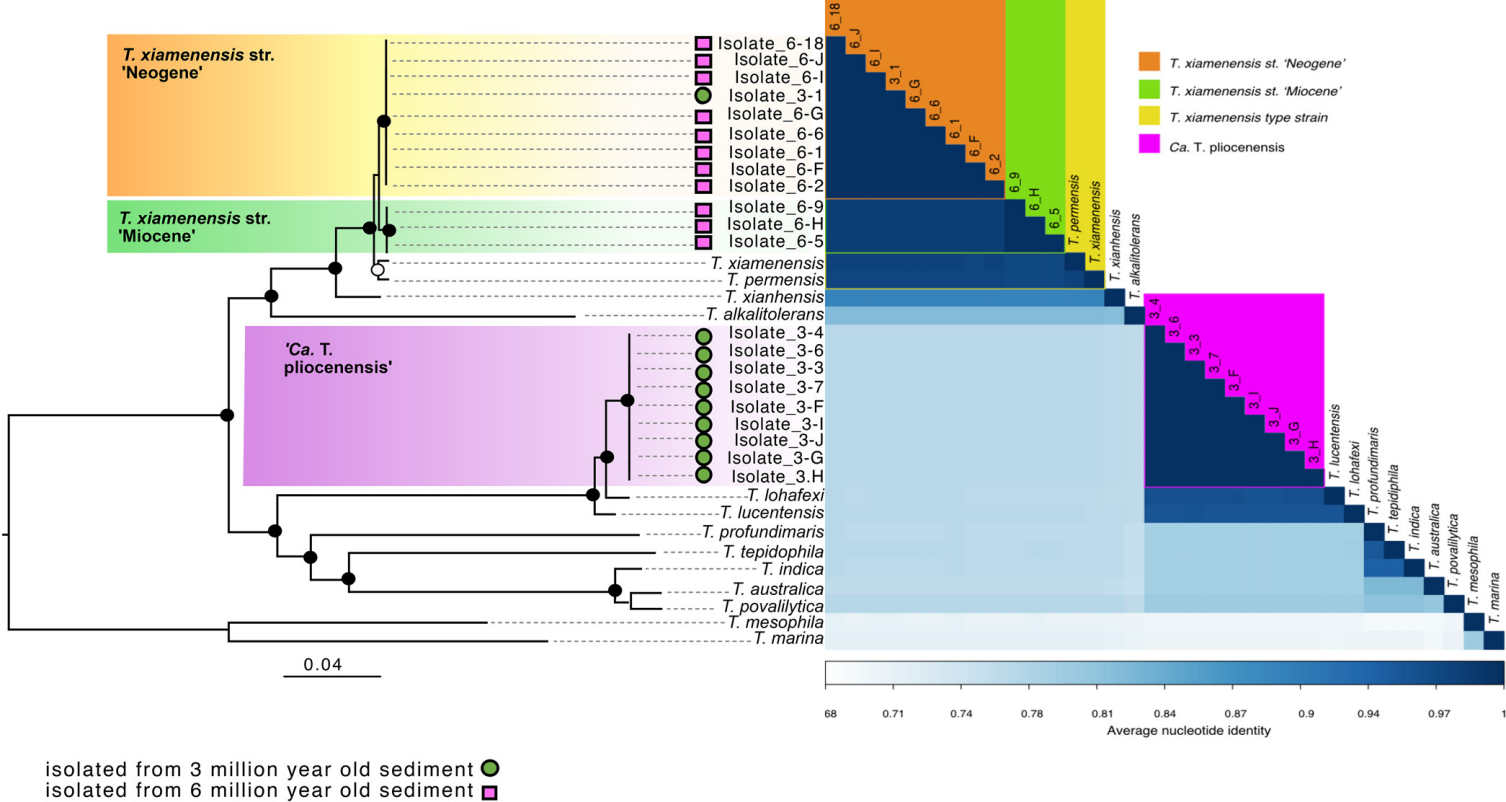
Average nucleotide identity (ANI) and core genome phylogeny. The tree is based on maximum likelihood and a concatenated alignment of 1,809 core genes. Black, gray, and white dots at the nodes represent >90%, >70%, and >50% bootstrap support, respectively.

Phylogenetic analysis of the full-length 16S rRNA gene sequences from the cultured isolates recovered two clades of subseafloor *Thalassospira*, affiliated with T. xiamenensis and T. lohafexi, respectively (Fig. 2C). However, phylogenomic reconstruction of the core genomes of *Thalassospira* type strains and the newly isolated subseafloor strains (1,809 orthologous genes) resolves into three distinct clades of subseafloor *Thalassospira* isolates (Fig. 3). One clade shares 96 to 97% genome-wide average nucleotide identity (ANI) and 99% 16S rRNA gene sequence identity with *T. xiamenensis* and T. permensis (Fig. 3). We named isolates in this clade *T. xiamenensis* strain “Neogene,” after the Neogene eon (2.8 to 23 mya), which covers both estimated ages of the sediment (3 mya and 6 mya) from which the strains in this clade were isolated. The subseafloor genomes in this clade correspond to the 16S rRNA gene ASV1 detected in the *in situ* frozen sediment core samples (Fig. 2B and C). A second clade contains three isolates from 6-mbsf sediment, which shares 97% genome-wide ANI with *T. xiamenensis* and *T. permensis.* Since all isolates in this clade were recovered from ca. 6-mya sediment deposited during the Miocene eon (5.33 to 23 mya), we report them as *T. xiamenensis* strain “Miocene” (Fig. 3). *T. xiamenensis* strain Miocene shared 99% 16S rRNA gene sequence identity with these most closely related type strains. The subseafloor genomes in the Miocene clade also correspond to ASV1 detected in the *in situ* frozen sediment core samples (Fig. 2B and C).

A third clade contains subseafloor *Thalassospira* cultures isolated only from 3-mbsf sediment and shares 95 to 96% genome-wide ANI with T. lucentensis and *T. lohafexi* (Fig. 3) and 99% 16S rRNA gene sequence identity with *T. lohafexi*. The subseafloor genomes in this clade correspond to the 16S rRNA gene ASV2 detected in the *in situ* frozen sediment core samples (Fig. 2B and C). Based on the genetic distinctness of these isolates, we consider them to be a new candidate species, according to recently provided criteria based on genome-wide ANI (23). Because all isolates of this third clade were recovered from 3-mya sediment, we propose the candidate name “*Candidatus* Thalassospira pliocenensis,” named after the Pliocene age (2.58 to 5.33 mya) of the deep-sea clay from which they were isolated.

### Pangenome analysis.

**FIG 4:**
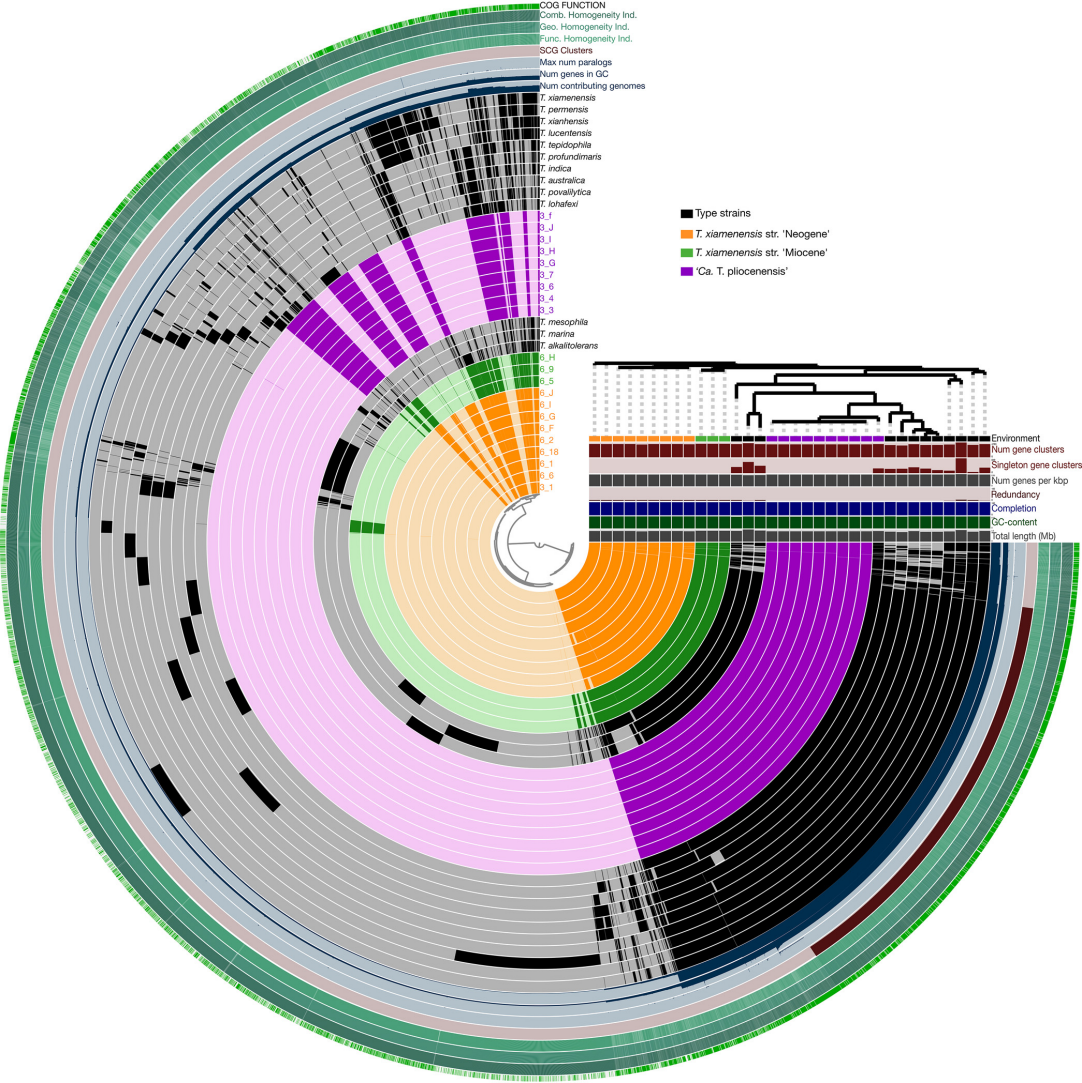
Pangenome analysis of all *Thalassospira* genomes included in the study. The internal dendrogram is an unweighted pair group method using average linkages (UPGMA) based on the presence/absence of shared gene orthologs. Black bars in the first (inner) 34 circles show the occurrence of gene clusters in the genomes of *Thalassospira* species. Gray areas and light colors in the circles represent gene clusters that were detected in the corresponding genomes. The next eight bars show statistics for the pangenome analysis of each gene cluster (inner circle to outer circle). The number of contributing genomes that have a hit in a gene cluster, GC content, the maximum number of paralogs, single-copy gene clusters (SCG), the functional homogeneity index, the geometric homogeneity index, the combined homogeneity index, and the presence of a Clusters of Orthologous Groups (COG) functional assignment are indicated. The categories on the right side (below the dendrogram) show the totals per genome for the number of gene clusters found in each genome and the number of genes per kilobase pair of genome. Redundancy, multiple occurrences of single-copy genes in a genome; completeness, calculated from the occurrences of single-copy gene sets in a genome.

The three subseafloor clades shared 38 to 41% of their genome content (Table S1). However, within each subseafloor clade, the proportion of gene content shared was much higher (>90%) (Fig. 4). This pangenome analysis shows substantial differences in the variable gene content across the three subseafloor clades, providing further evidence that each clade is a genetically distinct population (Fig. 4). Of the genes that are unique to the subseafloor genomes (e.g., core only to the subseafloor genomes), these represented 3 to 6% of the total gene content in the subseafloor genomes (Fig. 4), suggesting that they were acquired before burial. Furthermore, the fraction of the pangenome made up of singleton genes (seen in exactly one genome and no more) is 14% (±4%) of the flexible genome in the type strains (Fig. 4), which is significantly higher than the <0.1% in the subseafloor strains (*P* = 10^−7^ by a two-sided *t* test). The significantly higher numbers of singleton genes in the type strains are presumably recent acquisitions in the type strain genomes and consistent with a lack of recent gene gain in subseafloor genomes. The higher number of singleton genes in the flexible genomes of the type strains (Fig. 4) is consistent with the longer branch lengths of the type strains in the core genome phylogeny than those of the subseafloor strains (Fig. 3).

### Roles of mutation and recombination.

The ratio of nucleotide substitutions originating from mutations to those originating from homologous recombination (*r*/*m* ratio) can be used to measure the relative effect of homologous recombination on the genetic diversification of populations (24). Due to the physical isolation of individual bacterial cells, reduced cell concentrations, and the reduced availability of extracellular DNA for recombination in subseafloor sediments (25), we hypothesized that *r*/*m* ratios would be lower in the subseafloor *Thalassospira* populations than in the type strains. To test this, we used an established method, ClonalFrameML (26), to calculate the relative ratio of recombination to mutation (*R*/θ), the mean length of recombined DNA (δ), and the mean divergence of imported DNA (ν) for branch tips (existent genomes) and internal nodes (ancestral states) in the *Thalassospira* core genome phylogeny, which allows the calculation of *r*/*m* [*r*/*m* = (*R*/θ) × δ × ν]. This analysis showed that in the *Thalassospira* core genome, the *r*/*m* value is approximately 10 times lower in the subseafloor core genome (*r*/*m* ratio = 0.078) than in the type strains (*r/m* ratio = 0.71) (Table 1), indicating that homologous recombination plays a much lesser role in the diversification of the subseafloor strains. Because the *r*/*m* value estimated for the subseafloor isolates includes three different subseafloor clades, with an ANI differing up to 10% (Fig. 3), the *r*/*m* estimate for the subseafloor taxa should be reliable given that there is enough genetic variation to detect recombination. For example, the mean ν value between all of our subseafloor strains (ν = 0.026) (Table 1) is comparable to the values considered sufficient for detecting recombination (26).

**TABLE 1:** Contributions of recombination and mutation to nucleotide diversity in subseafloor populations^*a*^

Group	No. of strains	*R*/θ	δ	ν	*r*/*m* ratio	Avg no. of pseudogenes (SD)	Avg *dN*/*dS* ratio (SD)
All subseafloor and type strains	34	0.053	244	0.055	0.71	37 (±8)	0.025 (±0.011)
Type strains	13	0.04	333	0.053	0.71	21 (±3)	0.022 (±0.01)
Subseafloor strains	21	0.006	500	0.026	0.078	48 (±4)****	0.038 (±0.007)**

a The results from ClonalFrameML analysis were used to calculate the relative contributions of recombination and mutation in the core genome (*r*/*m*). *R*/θ, relative ratio of recombination compared to mutation; δ, average length of recombined (imported) DNA; ν, mean divergence of imported DNA. Also displayed are the average numbers of pseudogenes and *dN*/*dS* ratios (± standard deviations). **, *P* = 0.005 by a two-sided *t* test; ****, *P* = 0.000001 by a two-sided *t* test.

**FIG 5:**
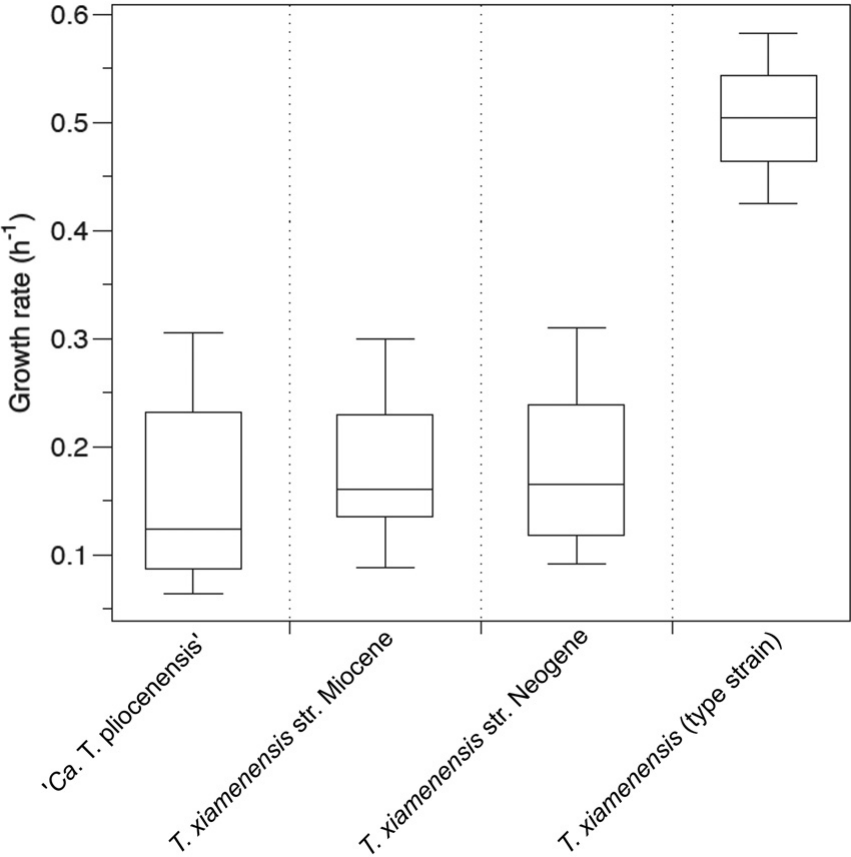
Laboratory growth rates of subsurface *Thalassospira* isolates in comparison to the *T. xiamenensis* type strain. Box plots illustrate interquartile ranges  ± 1.5× the interquartile range. The horizontal line in each box plot is the median. For subsurface strains, growth rates were calculated for each strain across three independent experiments. For the *T. xiamenensis* type strain, growth rates were calculated from triplicate flasks from one experiment.

**FIG 6:**
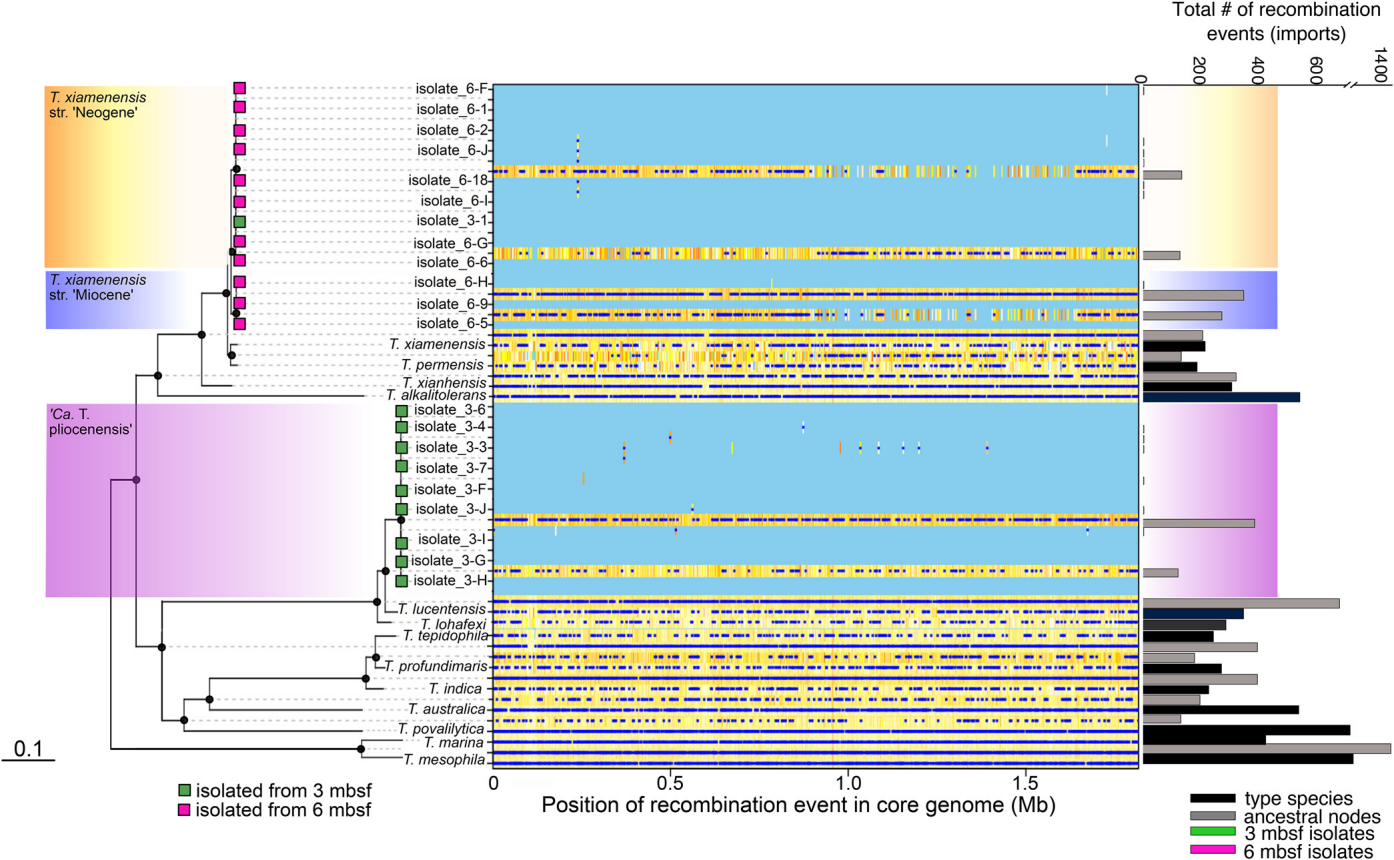
Recombination in the conserved core genome is limited in subseafloor *Thalassospira* populations. The maximum likelihood (PhyML) phylogenetic tree is based on a concatenated alignment of 1,809 genes conserved across all *Thalassospira* genomes (“core genes”). Black circles on nodes represent bootstrap values of >95%. The positions of recombination events in the core genome are represented by dark-blue dots. Positions of low nucleotide diversity and no recombination events in the core genome are shown in light blue. Nucleotide diversity at specific sites in the core genome is illustrated with a color gradient (white, less diversity; orange, more diversity). Histograms on the right display the total number of recombination events (imports) in each genome sequence and ancestral-state reconstructions (internal nodes), as detected by ClonalFrameML.

Laboratory growth rates of the subseafloor *Thalassospira* isolates ranged from 0.064 to 0.31 h^−1^, which were significantly lower than that of the type strain of *T. xiamenensis* (Fig. 5). These lower growth rates than that of the “surface-world” type strain are consistent with the *r*/*m* values of the subseafloor *Thalassospira* isolates (*r/m* = 0.078) being anomalously low compared to those of free-living bacteria isolated from the surface world, which have *r*/*m* values that range from 0.1 to 64 (24). Concomitant with the 10-fold-lower *r*/*m* values than those of the type strains (Table 1), the subseafloor *Thalassospira* core genomes exhibit far fewer inferred recombination events with imported DNA than the *Thalassospira* type strains and the ancestral states of the last common ancestors shared with the type strains (Fig. 6).

The relatively low levels of recombination in the genomes of the subseafloor isolates, compared to the type strains, is consistent with a shift away from a recombination-influenced population toward a more clonal population structure since the subseafloor *Thalassospira* strains diverged from the last common ancestor shared with the type strains. This finding is supported by the pangenome analysis, which shows a significantly (*P* = 10^−7^ by a two-sided *t* test) lower number of singleton genes in the subseafloor strains than in the type strains (Fig. 4), indicating a lack of recent gene gain in subseafloor genomes compared to the type strains. Clonal growth over millions of years appears to have resulted in a phenotypic change given the relatively lower growth rates of the subseafloor strains (Fig. 5).

We looked for evidence of evolution in the subseafloor strains by investigating the number of pairwise nucleotide-level differences in the subseafloor *Thalassospira* genomes. We identified tens to thousands of differences within each subseafloor *Thalassospira* clade (Fig. S2). The differences in subseafloor *Thalassospira* strains were present in a clade-specific manner (Fig. S3) and consisted of SNPs and insertion-deletion (indel) events. Most (>90%) within-clade differences were represented by indels across all three clades, and all intraclade differences in *T. xiamenensis* strain Miocene were represented by indels (Fig. S3). While the precise chromosomal locations of SNPs and indels are clade specific, the SNPs and indels often occurred in genes coding for related functions across the different clades. These included indels within genes with predicted annotations involved in flagellar motility (*flhB*, *flhL*, *fliQ*, and *fliM*), transcription (*tetR* and *fis* family transcriptional regulators), cell wall biogenesis (peptidase S41 and peptidoglycan dd-metalloendopeptidase M23), and the transport and metabolism of amino acids and carbohydrates (Fig. S3). This suggests that although the three *Thalassospira* lineages are evolving independently, the shared environmental setting renders similar traits superfluous across populations and susceptible to genetic changes or loss.

10.1128/mBio.01150-21.2 FIG S2 Nucleotide diversity (SNPs and regions with indels) between pairs of subseafloor *Thalassospira* genomes. Download FIG S2, PDF file, 0.2 MB. Copyright © 2021 Orsi et al. 2021 Orsi et al. https://creativecommons.org/licenses/by/4.0/ This content is distributed under the terms of the Creative Commons Attribution 4.0 International license.

10.1128/mBio.01150-21.3 FIG S3 Numbers of interpopulation SNPs and indels at different positions in the core genome alignment for each of the three subseafloor populations. The gene annotations for the corresponding regions are shown. Download FIG S3, PDF file, 0.05 MB. Copyright © 2021 Orsi et al. 2021 Orsi et al. https://creativecommons.org/licenses/by/4.0/ This content is distributed under the terms of the Creative Commons Attribution 4.0 International license.

We considered the possibility that the observed nucleotide substitutions and indels occurred within the subseafloor populations during the culture enrichment and cultivation processes. The generation times of *Thalassospira* (OTU_6) in the incubation measured by quantitative PCR (qPCR) (36 ± 1.5 days [mean ± SD]) indicate an estimated maximum of 15.7 doublings over the enrichment (Fig. S1). Approximately 63 additional generations may be attributable to subsequent cultivation prior to genome sequencing (Fig. S1). At common bacterial mutation or chromosomal deletion rates of 10^−9^ to 10^−10^ mutations bp^−1^ generation^−1^ (27, 28), more than 4,000 generations would be required to obtain the observed nucleotide diversity present in the subsurface *Thalassospira* genomes (Fig. S4). Thus, newly accumulated nucleotide changes in culture are likely insufficient to explain the observed interpopulation nucleotide diversity between the subseafloor genomes (Fig. S4). While it is possible that the observed clonal population structure resulted from the 18-month enrichment, the genomes that we sequenced were not identical, even within clades, which might be expected if clonality was the result of enrichment. For example, the number of pairwise nucleotide differences (including indels and SNPs) in the core genomes of each clade ranged from 0 bp (3_G and 3_H) to 3,602 bp (Fig. S2). At a mutation or deletion rate of 10^−9^ per generation, between 10^3^ and 10^4^ generations would need to accrue to attain this observed genetic diversity (Fig. S4).

10.1128/mBio.01150-21.4 FIG S4 Interpopulation nucleotide diversity likely did not arise during laboratory cultivation. (A) Model of the nucleotide diversity expected per genome (4.7 Mbp) as a function of the total number of generations at five different nucleotide divergence rates. Shading depicts the range of observed nucleotide diversity in subseafloor *Thalassospira* genomes (nucleotide diversity includes numbers of SNPs and indels) as described below for panel B. (B) Distribution of interpopulation nucleotide diversity. Points are the numbers of pairwise SNPs detected in subseafloor *Thalassospira* genomes. Box plots illustrate interquartile ranges ± 1.5× the interquartile range. The horizontal line in each box plot is the median. Download FIG S4, PDF file, 0.1 MB. Copyright © 2021 Orsi et al. 2021 Orsi et al. https://creativecommons.org/licenses/by/4.0/ This content is distributed under the terms of the Creative Commons Attribution 4.0 International license.

### Substitutions and pseudogenes are fixed in subseafloor populations.

**FIG 7:**
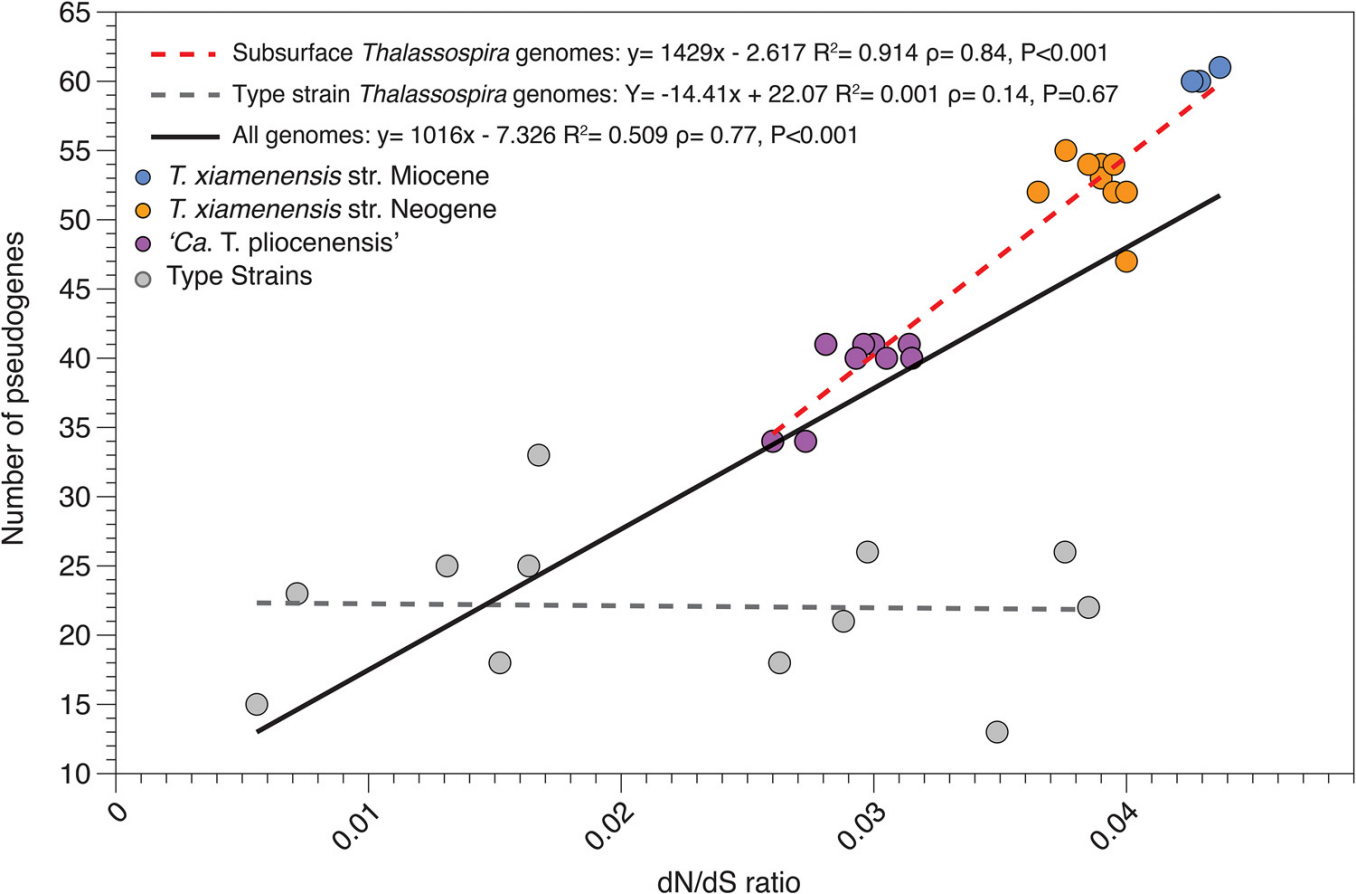
The numbers of pseudogenes and *dN*/*dS* ratios are elevated and correlated in subseafloor *Thalassospira* populations. Subseafloor genomes accumulate more pseudogenes as a function of increasing *dN*/*dS* ratios than the type strains. Linear regressions for type strains, subseafloor strains, and all strains are displayed.

Compared to the type strains, the subseafloor *Thalassospira* genomes exhibit high numbers of pseudogenes (nonfunctional parts of the genome that resemble functional genes) and nonsynonymous substitutions (substitutions that alter the amino acid sequence of a protein). We identified 47.9 ± 8.57 pseudogenes (mean ± SD) in the genomes of subseafloor *Thalassospira* isolates, which is significantly higher than the number of pseudogenes identified in the type strains (22.1 ± 5.52 pseudogenes [mean ± SD]) (Table 1, Fig. 7, and Fig. S1) (*P* = 1.5E−10 by a two-sided *t* test). We investigated whether the genomes from the subseafloor *Thalassospira* isolates exhibited elevated ratios of nonsynonymous to synonymous substitutions (*dN*/*dS* ratios) that might further indicate evolution in clonal populations and relaxed purifying selection across the genome. Similarly, we observed a modest but significant elevation of genome-wide *dN*/*dS* ratios in the genomes of the subseafloor *Thalassospira* strains (0.035 ± 0.006 [mean ± SD]) relative to the type strains (0.022 ± 0.012 [mean ± SD]) (*P* = 0.0002 by a two-sided *t* test) (Table 1, Fig. 7, Fig. S1, and Table S2). While the increased *dN*/*dS* ratio is consistent with relaxed purifying selection, such small changes are possibly not biologically or evolutionarily meaningful. Additionally, the calculation of *dN*/*dS* ratios may be confounded by the high similarity of the subseafloor genomes to each other and to the type strains (see Materials and Methods).

10.1128/mBio.01150-21.7 TABLE S2 Summary of aBSREL results. aBSREL is based on the in-frame codon-concatenated core genome alignment. Each run included one representative from each of the three subseafloor clades and all (run 1) or a subset (runs 2 to 10) of the type strains. Black filling indicates that the genome was not included in the run. Highly similar genomes result in unreliable *dN*/*dS* results, and thus, aBSREL had to be run 10 separate times with different (highly similar) genomes from the subseafloor clades included separately. The average *dN*/*dS* ratio of the type strains (0.023 ± 0.011) was significantly lower than that of the subseafloor strains (0.035 ± 0.006) (*P* = 0.00005 by a 2-sided *t* test). Download Table S2, XLSX file, 0.01 MB. Copyright © 2021 Orsi et al. 2021 Orsi et al. https://creativecommons.org/licenses/by/4.0/ This content is distributed under the terms of the Creative Commons Attribution 4.0 International license.

**FIG 8:**
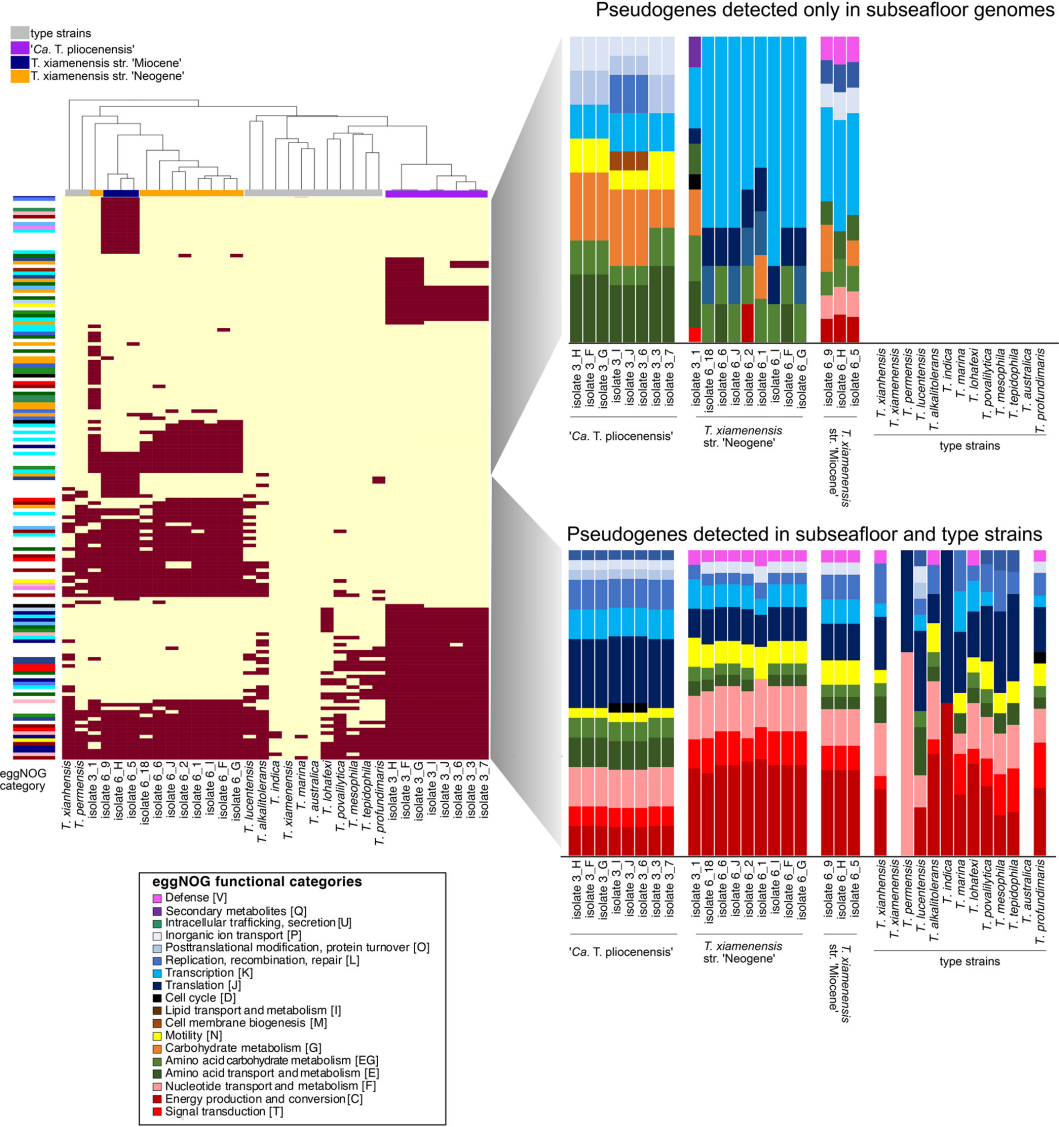
Heat map showing the presence/absence of pseudogenes in the subseafloor genomes and the presence of these pseudogenes in type strains. Functional annotations (against eggNOG) of pseudogenes found in the subseafloor genomes only are compared to functional annotations of pseudogenes found in both the subseafloor and type strains. The dendrogram is a UPGMA clustering analysis based on pseudogene presence/absence in the heat map.

**FIG 9:**
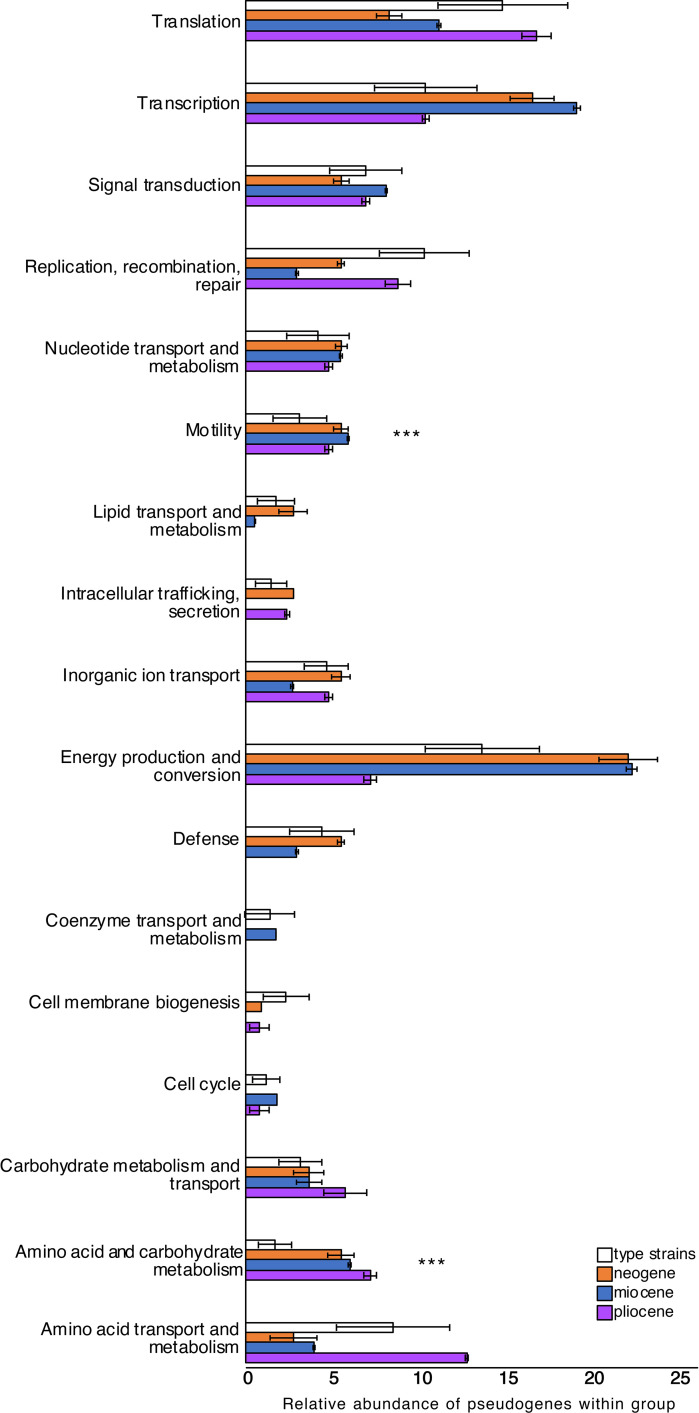
Histograms showing the average relative abundances of functional categories of pseudogenes found within each of the three subseafloor populations, compared to the type strains. The error bars represent standard deviations, and asterisks indicate abundances of functional categories of pseudogenes that were significantly higher in the subseafloor genomes than in the type strain genomes (*P* < 0.01 by a two-sided *t* test).

Similar to the nucleotide differences (SNPs and indels) (Fig. S3), the composition of pseudogenes occurred in a clade-specific manner (*P* = 0.001 by analysis of similarity [ANOSIM]) (Fig. 8). Compared to the type strains, the predicted annotations of the pseudogenes in the subseafloor *Thalassospira* genomes are skewed toward those involved in transcription, energy conservation, amino acid and carbohydrate metabolism, and flagellar motility (Fig. 8). Moreover, subseafloor genomes have significantly higher numbers of pseudogenes involved in motility (flagellar biosynthesis genes *fliN*, *fliK*, and *flhO*) than the type strains (*P* = 0.003 by a two-sided *t* test) (Fig. 9). These flagellar biosynthesis genes are different from the flagellar biosynthesis genes containing intrapopulation indels (*fliQ*, *fliK*, *flhB*, *flgH*, and *flgL*) (Fig. S3), but the pseudogenized *fliN* and *fliK* genes are syntenic with the indel-containing flagellar genes (Fig. S3), indicating that both indels and pseudogenization have targeted different genes within the flagellar operon.

Burial in the sediment millions of years ago resulted in the physical isolation of subsurface *Thalassospira* cells and was the impetus for a transition from freely recombining populations with a large *N_e_* to recombination-limited clonal populations with a small *N_e_* that rarely encounter genetically diverse recombination partners that introduce genetic diversity into the population. The combined effects of physical isolation, low cell concentrations, and little or no motility due to low permeability and energy limitation further reduced recombination events. These extreme physical conditions resulted in clonal subseafloor *Thalassospira* populations that are characterized by the inability to overwrite deleterious mutations through recombination. Our observations of slightly elevated *dN*/*dS* ratios and an increased number of pseudogenes (Fig. 7) are evidence the subsurface microbes are experiencing variability in selection, changes that are more likely to be fixed in the absence of homologous recombination. However, because of the small magnitude of the changes, it is challenging to determine whether these observations result from the fixation of deleterious mutations via genetic drift, as is commonly observed in endosymbiotic bacteria (29), or variation in selective pressures resulting from the environmental changes that accompanied burial. For example, some functions appear to be more prone to gene decay than others, such as those involved in flagellar motility. We identified both indels (*fliQ*, *fliK*, *flhB*, *flgH*, and *flgL*) (Fig. S3) and pseudogenes (*fliN*, *fliK*, and *flhQ*) (Fig. 8 and 9) in flagellar operons, suggesting that flagellar motility was a slightly beneficial trait in *Thalassospira* populations before burial but has become a nonessential trait subject to relaxed selection in abyssal clays characterized by very low permeability and an extremely small pore diameter despite their high porosity (18). This observation is further supported by the apparent convergent gene decay in the flagellar biosynthesis operon across the Miocene and Neogene strains (Fig. S5).

10.1128/mBio.01150-21.5 FIG S5 Intraclade SNPs and indels within the flagellar assembly operon. In the synteny plot (top), green-highlighted genes are those that have indels within both the “Neogene” and “Miocene” clades of *T. xiamenensis*. The orange genes are those that have only SNPs or indels between members of the Neogene clade. The bottom panel shows the regions of the gene alignments where the positions of the intraclade indels and SNPs are located. Download FIG S5, PDF file, 0.2 MB. Copyright © 2021 Orsi et al. 2021 Orsi et al. https://creativecommons.org/licenses/by/4.0/ This content is distributed under the terms of the Creative Commons Attribution 4.0 International license.

### Outlook.

Our findings demonstrate that the subseafloor *Thalassospira* genomes analyzed here have evolved within clonal populations that have experienced a reduction in homologous recombination. Clonal populations can become subject to genetic drift whereby deleterious mutations become fixed and Muller’s ratchet (30) eventually leads to the extinction of endosymbiotic bacterial lineages (29). Our genomes show similar signs of evolution, but the *dN*/*dS* ratios observed in the subseafloor *Thalassospira* genomes are much lower than those observed in endosymbiotic bacteria (31), and subsequent genome reduction was absent. We speculate that the subseafloor *Thalassospira* isolates have not experienced sufficient generations to observe large changes in *dN*/*dS* ratios or genome reduction given that perpetual and severe energy limitation results in increased generation times with depth below the seafloor (2–7).

Subseafloor cell concentrations are low and decrease substantially with increasing depth (1). Because genetic drift has a stronger effect on populations with a small *N_e_* (29), physically isolated microbes experiencing clonal growth and reduced homologous recombination in the deep biosphere may be particularly prone to genetic drift-mediated evolution. Because the subseafloor biosphere contains a large fraction of all bacterial cells on Earth (1), our findings suggest that drift-like evolutionary processes in the absence of homologous recombination may be much more widely distributed in nature than previously thought. Future assessments of homologous recombination and drift in single-cell genomes from uncultured lineages of bacteria and archaea that comprise most subsurface energy-limited communities (32) could be used to assess how widespread this evolutionary mechanism is within the subsurface biosphere.

## MATERIALS AND METHODS

### Sampling and pore water chemistry.

All samples were taken during expedition KN223 on the *R/V Knorr* in the North Atlantic, from 26 October to 3 December 2014, at site 11 (22°47.0′N, 56°31.0′W; water depth of ∼5,600 m), via a long-core piston-coring device (∼28 m). Additional details of sampling have been described previously (33). Dissolved oxygen concentrations in the core sections were measured with optical O_2_ sensors from the equilibrated core sections and measured with needle-shaped optical O_2_ sensors (optodes) (PreSens, Regensburg, Germany) as described previously (33). The dissolved-O_2_ data from expedition KN223 are archived and available in the Integrated Earth Data Applications (IEDA) database (https://www.nodc.noaa.gov/archive/arc0127/0178259/2.2/data/0-data/KN223_603663_r2rnav/).

### Physical sediment properties.

Deep-sea abyssal clay is characterized by very low permeability and an extremely small pore diameter despite its high porosity (18). Deep-sea clay particles have a grain size of <0.2 μm, and the pore space between clay particles is smaller than a bacterial cell, limiting the movement of bacteria through pore space in the clay. Bioturbation can vertically redistribute cells within marine sediment, but bioturbation is restricted to the upper 0.5 m of sediment (34) and thus cannot vertically transport sediment surface material and microbes to depths of 3 and 6 mbsf. Considering the mean sedimentation rate of 1 m per million years, it can be concluded that the bacterial cultures obtained from sediment collected at 3 and 6 mbsf have been physically isolated from the surface world for millions of years.

### DNA extraction, qPCR, and 16S rRNA gene sequencing.

DNA extractions, qPCR, and 16S rRNA gene sequencing were described previously by Vuillemin et al. (17). In brief, subcores were sampled aseptically with sterile syringes in a UV-sterilized DNA/RNA clean HEPA-filtered laminar flow hood. DNA was extracted from 10 g of sediment transferred into 50 ml of lysing matrix E tubes (MP Biomedicals) containing silica glass beads and homogenized for 40 s at 6 m/s using a FastPrep-24 5G homogenizer (MP Biomedicals) in the presence of 15 ml of preheated (65°C) sterile-filtered extraction buffer (76 vol% 1 M NaPO_4_ [pH 8], 15 vol% 200-proof ethanol, 8 vol% MoBio lysis buffer solution C1, and 1 vol% SDS). The samples were incubated at 99°C for 2 min, frozen overnight at −20°C, thawed, and frozen again at −20°C overnight, followed by an additional incubation at 99°C for 2 min and a second homogenization step using the settings described above. After the second homogenization, the samples were centrifuged for 15 min, and the supernatants were concentrated to a volume of 100 μl using 50-kDa Amicon centrifugal filters (Millipore). Coextracted PCR-inhibiting humic acids and other compounds were removed from the concentrated extract using the PowerClean Pro DNA cleanup kit (MoBio). Extraction blanks were included alongside the samples to assess laboratory contamination during the extraction process.

DNA was quantified fluorometrically using a Qubit system with a double-stranded DNA (dsDNA) high-sensitivity kit (Life Technologies). DNA templates were used for qPCR amplifications with updated 16S rRNA gene primer pair 515F (5′-GTG YCA GCM GCC GCG GTA A-3′) and 806R (5′-GGA CTA CNV GGG TWT CTA AT-3′) as previously described (35). All qPCRs were set up in 20-μl volumes with 4 μl of the DNA template, 20 μl of SsoAdvanced SYBR green supermix (Bio-Rad), 4.8 μl of nuclease-free H_2_O (Roche), 0.4 μl of primers (10 μM; biomers.net), and 0.4 μl of MgCl_2_ and carried out on a CFX-Connect qPCR machine (Bio-Rad) for gene quantification. For 16S rRNA genes, we ran 40 PCR cycles of two steps corresponding to denaturation at 95°C for 15 s and annealing and extension at 55°C for 30 s. All qPCRs were set up in 20-μl volumes with 4 μl of the DNA template. Gel-purified amplicons were quantified in triplicate using QuantiT dsDNA reagent (Life Technologies) and used as a standard. An EpMotion 5070 automated liquid handler (Eppendorf) was used to set up all qPCRs and prepare the standard curve dilution series spanning from 10^7^ to 10^1^ gene copies. Reaction efficiency values in all qPCR assays were between 90% and 110% with *R*^2^ values of >0.95% for the standards. Barcoded PCR for Illumina sequencing was performed using the custom primer dual-indexed approach that targets the V4 hypervariable region of the 16S rRNA gene using dual-indexed 16S rRNA gene primer pair 515F (5′-GTGYCAGCMGCCGCGGTAA-3′)/806R (GGACTACNVGGGTWTCTAAT) (35). Barcoded V4 hypervariable regions of amplified 16S rRNA genes were sequenced on an Illumina MiniSeq platform according to a previously established protocol (35). Bioinformatic processing of these previously published sequence data was described previously by Vuillemin et al. (17) in detail.

### Long-term incubation setup.

Prior to setting up the incubations, the subcores were sampled with sterile syringes using the sample aseptic technique used for DNA extraction. For each sample depth, 7 g of abyssal clay was placed into sterile 20-ml glass flasks and incubated with 4 ml of sterile artificial seawater composed of either H_2_^18^O (97% atomic enrichment) or unlabeled artificial seawater. Vials were crimp sealed, with an oxygenated headspace of approximately 10 ml, and incubated at 8°C. The artificial seawater was different from the porewater at depth because there was no added nitrate, but there was also no added ammonia, which should be similar to *in situ* conditions where ammonia is generally below the limit of detection (17). Oxygen was measured continuously throughout the incubations using noninvasive fiber-optic measurements as described previously (36). Small fluctuations in the oxygen measurements in the killed-control and experimental incubations were likely due to temperature fluctuations of the incubator itself (±1°C) since the noninvasive fiber-optic oxygen sensor spots are temperature sensitive (36). Oxygen consumption was detectable over 18 months in slurries consisting of sediment and sterile artificial seawater (see Fig. S1 in the supplemental material), suggesting the presence of actively respiring microbes. The rate of oxygen consumption was calculated by subtracting the starting concentration of O_2_ from the final concentration of O_2_ and dividing this by the number of days for the incubation.

We used quantitative stable isotope probing (qSIP) to measure the atoms percent ^18^O enrichment of actively growing microbial taxa as described previously (17, 37). In brief, after 7- and 18-month incubations, DNA was extracted and subjected to cesium chloride (CsCl) density gradient centrifugation. The same 16S rRNA gene 515F/806R primers (described above) were used for qPCR (described above) to determine density shifts in the peak DNA of buoyant density (BD) for each incubation. 16S rRNA gene amplicons from each fraction resulting from density gradient fractionation were Illumina sequenced as described previously (17). To identify contaminants that may have entered during the fractionation process, we also included in the sequencing run extraction blanks from the SIP fractionation. OTUs containing sequences from extraction blanks were removed. Excess atoms percent ^18^O enrichment were calculated for each OTU (including OTU_6, corresponding to the subseafloor *Thalassospira* strains) according to the equations for quantifying per-OTU atomic enrichment.

The number of doublings for the *Thalassospira* OTU (OTU_6) detected at the 18-month time point was calculated using the qPCR-normalized relative abundance of the 16S rRNA genes at 0 and 18 months. The number of generations was calculated as *X* = 2*^n^X*_0_, Where *X* and *X*_0_ are the final and initial cell densities, respectively, and *n* is the number of generations.

### Enrichments, cultivation, and subcultivation.

After 18 months of incubation in sterile ^18^O-labeled artificial seawater, 25 μl of the slurry was plated onto solid medium (10 mg/ml yeast extract and 8 mg/ml agar in artificial seawater [30 mM MgCl_2_·6H_2_O, 16 mM MgSO_4_·7H_2_O, 2 mM NaCO_3_, 10 mM KCl, 9 mM CaCl_2_, 450 mM NaCl]), and after 2 days of incubation in the dark at room temperature, abundant colonies were observed growing on the surface of the petri dishes (Fig. S1). No colonies were observed to grow on control petri dishes that received 25 μl of the ^18^O-labeled artificial seawater slurry incubated for 18 months using starting material from the autoclaved sediment (killed controls). This indicated that the colony-forming bacteria were from the sediment and not contaminants introduced during the experimental setup of the incubations. We attempted to culture chemoheterotrophic microbes directly from the collected sediment samples under the same conditions, but no CFU were observed on the petri dishes, even after several months of incubation. Thus, long-term incubation of the sediment at 8°C simply in the presence of added water apparently stimulated the activity of many subseafloor bacteria to a point at which they were able to grow on the surface of a petri dish.

A total of 21 colonies were picked from petri dishes containing colonies from the 3-mbsf and 6-mbsf slurries (10 colonies picked from the 3-mbsf slurry and 11 colonies picked from the 6-mbsf slurry). These colonies were streaked individually onto separate new petri dishes, and a single colony was picked from each of the separate petri dishes (representing the original CFU from the enrichment) and grown in sterile liquid medium (10 mg/ml yeast extract and 8 mg/ml agar in artificial seawater [30 mM MgCl_2_·6H_2_O, 16 mM MgSO_4_·7H_2_O, 2 mM NaCO_3_, 10 mM KCl, 9 mM CaCl_2_, 450 mM NaCl]). Single colonies were then grown in liquid medium. A portion of each of these colonies was used for DNA extraction and genome sequencing, and the remaining volume was frozen as glycerol stocks. Growth rates were determined in experiments with 20-ml crimp-sealed glass flasks containing 0.1 ml of the glycerol stock inoculated into 10 ml liquid medium (10 mg/ml yeast extract in artificial seawater [30 mM MgCl_2_·6H_2_O, 16 mM MgSO_4_·7H_2_O, 2 mM NaCO_3_, 10 mM KCl, 9 mM CaCl_2_, 450 mM NaCl]) with a 10-ml headspace and gentle shaking. The optical density (OD) at 600 nm was measured once every 30 min with a spectrophotometer, and growth rates were calculated from the exponential phase using the formula μ = [ln(*X*) − ln(*X*_0_)]/(*t* − *t*_0_), where *X* and *X*_0_ are the final and initial optical densities (at 600 nm), respectively, and *t* and *t*_0_ are the times at which those optical densities were made (final and initial, respectively). Under these conditions, the growth rates of the subseafloor *Thalassospira* strains were similar across all three clades and ranged from 0.064 to 0.31 h^−1^ (Fig. S5). The growth experiments for all subseafloor and type strains were performed alongside one another in the same bacteriological growth medium formulation, at the same temperature, in identically constructed and prepared flasks, and at the same shaking speed.

### Assessing the possibility of genome evolution during the 18-month enrichment.

Because bacteria can evolve on laboratory experimental timescales (10, 11), we considered the possibility that all diversification and evolution happened during the 1.5-year enrichment. Using the qPCR-based estimate of the doubling time of the subseafloor *Thalassospira* OTU (OTU_6) in the incubation, which was 36 (±1.5) days, the number of doublings with this rate over this time period would be 15.67. After the enrichment, we spread cells onto petri plates. Assuming that each colony attained 3.3E9 cells per colony (38) from a single cell, we calculate another 31.62 generations per colony (using *X* = 2*^n^X*_0_), for a total of 47.29 (15.67 + 31.62 = 47.29) generations per isolate; repeating this isolation with a single cell and colony formation a second time prior to genome sequencing, we arrive at 47.29 + 31.62 = 78.91 generations. According to “Drake’s rule” (28), bacteria experience on average 1 mutation per 300 genomes replicated; thus, the amount of nucleotide diversity (tens to thousands of nucleotide differences) (Fig. S2) that could be accumulated during the incubation is insufficient to explain the observed divergence among the three subseafloor populations. We thus conclude that the interpopulation nucleotide diversity resulted from mutations that were acquired after they were buried. We acknowledge that some cells likely did not survive during the 18-month incubation, which may cause an underestimation of the number of generations. Moreover, upon growth in a colony, additional numbers of generations are passed. Therefore, while our calculated number of generations is an underestimate, we are within an order of magnitude of the real value.

### Genome sequencing, *de novo* assembly, and annotation.

DNA was extracted from the isolates grown in liquid culture until the end of exponential phase as described above. After reaching stationary phase, cultures were pelleted via centrifugation, and the supernatant was decanted. The cell pellets were resuspended in a preheated (65°C) sterile-filtered extraction buffer (76 volume % 1 M NaPO_4_ [pH 8], 15 volume % 200-proof ethanol, 8 volume % MoBio lysis buffer solution C1, and 1 volume % SDS), added to lysing matrix E tubes (MP Biomedicals) containing silica glass beads, and homogenized for 40 s at 6 m/s using a FastPrep-24 5G homogenizer (MP Biomedicals). The samples were centrifuged for 15 min, and the dissolved high-molecular-weight DNA in the supernatant was concentrated to a volume of 100 μl using 50-kDa Amicon centrifugal filters (Millipore). The concentrated extract was cleaned of proteins and other nongenomic DNA organic matter using the PowerClean Pro DNA cleanup kit (MoBio). Extraction blanks were added alongside the samples to assess laboratory contamination during the extraction process. Genomic libraries were prepared using the Nextera XT DNA library prep kit (Illumina). Quality control and quantification of the libraries were performed on an Agilent 2100 Bioanalyzer system using high-sensitivity DNA reagents and DNA chips (Agilent Genomics). Genomic libraries were sequenced to a depth of ca. 100× coverage using a high-output paired-end 2-by-150 sequencing reagent kit (Illumina).

In addition to Illumina sequencing, the high-molecular-weight genomic DNA was sequenced using the Nanopore MinION system. Sequencing libraries for the MinION system were prepared using the ligation sequencing kit (Oxford Nanopore Technologies) according to the manufacturer’s instructions. Barcoded libraries were sequenced on the MinION system using a Flongle R9 flow cell and base called and demultiplexed using the MinIT system with ont-minit-release v19.12.5 and ont-guppy-for-minit v3.2.10 for base calling (Oxford Nanopore Technologies).

A hybrid assembly was performed using both the short (Illumina)- and long (Nanopore)-read sequencing data using Unicycler (v.0.4.0), which uses *de novo*-assembled Illumina data from SPADES to polish the *de novo*-assembled contigs obtained from Nanopore data using RACON (39). We used the default settings available in Unicycler to coassemble the Illumina data (forward and reverse fastq files) and Nanopore data (single fastq file), which consisted of the following commands: ./unicycler -1 isolate-6-1_illumina_R1_001.fastq -2 isolate-6-1_illumina_R2_001.fastq -l isolate-6-1_Nanopore.fastq –spades_path ./SPAdes-3.13.0-Darwin/bin/spades.py –racon_path ./racon/build/bin/racon –bowtie2_build_path ./bowtie2-build –bowtie2_path ./bowtie2 -o out. The combined assemblies of Illumina and Nanopore data resulted in a relatively low number of contigs (9 to 12 per genome) and a predicted genome completeness of 100% for nearly all genomes (Table S1). Genome completeness was determined using CheckM (40), using the family *Rhodospirillaceae* as the reference group (which contains all species within the genus *Thalassospira*). Genomes were annotated using RASTk (41).

### Core genome phylogenetic analyses.

The core genome was defined as the set of orthologous genes that were shared in all subseafloor and “type strain” *Thalassospira* genomes. NCBI genome accession numbers for the type strain genomes of *Thalassospira* (those isolated from surface-world habitats) are as follows: NZ_JAATJD010000001.1 for *T. tepidiphila*, NZ_JPWA00000000.1 for *T. xianhensis*, NZ_ATWN01000001.1 for *T. lucentensis*, NZ_FTON01000032.1 for *T. xiamenensis*, NZ_AMRN01000001.1 for *T. profundimaris*, NZ_FNTU01000002.1 for *T. permensis*, NZ_JFKB00000000.1 for *T. alkalitolerans*, NZ_CP031555.1 for *T. indica*, NZ_CP024199.1 for *T. marina*, NZ_PGTS00000000.1 for *T. povalilytica*, NZ_NXGX00000000.1 for *T. lohafexi*, NZ_JFKA00000000.1 for *T. mesophila*, and NZ_JRJE00000000.1 for *T. australica*. Orthologous genes between the subseafloor and type strains were defined as those sharing >30% amino acid similarity to the collective suite of genes within the type strain Thalassospira xiamenensis M-5. *T. xiamenensis* M-5 was chosen as the reference genome for this purpose because it is the only publicly available genome of a cultivated *Thalassospira* isolate that is completely closed and represents a single chromosome and a 190-kb plasmid. A total of 1,809 orthologous genes (defined as being present as only a single copy in each genome) were identified that are carried by all *Thalassospira* strains that had >30% sequence similarity to genes within the *T. xiamenensis* M-5 genome. We define these genes as orthologs, as opposed to paralogs, because they are present as only a single copy in each genome. Paralogs were identified in *Thalassospira* genomes by performing similarity searches for genes (BLASTp queries) that had similarity to a *T. xiamenensis* gene (BLASTp subjects) and then removing subjects (potential core genes) that had hits from multiple queries from the same query genome. Only *T. xiamenensis* genes (BLASTp subjects) that had similarity from a single BLASTp query across all *Thalassospira* strains were kept as orthologs. This should select only single-copy genes (genes that have not been duplicated within the genomes). Because the genes have homology between the different genomes and are present as a single copy across all genomes compared, we interpret them to be orthologs. Each of these 1,809 orthologous genes was individually aligned between all *Thalassospira* strains using MUSCLE (42), and the 1,809 individual alignments were then concatenated into a single core genome alignment for the subsequent phylogenomic analysis using Geneious Prime (version 2019.2.1). After the concatenation of all core genes, the total size of the core genome alignment was 1,817,073 nucleotide characters, with 34 taxa (21 subseafloor strain and 13 type strain taxa). A maximum likelihood phylogeny was created using PhyML (43) with a generalized time-reversible (GTR) model of evolution and 100 bootstrap replicates, which was implemented in SeaView (44). The resulting phylogenetic tree and the concatenated core genome alignment were used as inputs for subsequent ClonalFrameML and *dN*/*dS* ratio (ratio of nonsynonymous to synonymous mutations) analyses. Full-length 16S rRNA gene sequences were amplified from the isolated subseafloor bacteria from DNA extracted from cell pellets (using the protocol described above) using PCR (30 cycles of 95°C for 30 s, 60°C for 30 s, and 72°C for 2 min) with universal PCR primers 27F (5′-AGAGTTTGATCMTGGCTCAG-3′) (45) and 1391R (5′-GACGGGCGGTGTGTRCA-3′) (46) and Sanger sequenced, as a quality control measure. Quality control and assembly of the Sanger-sequenced 16S genes (forward and reverse sequences, each ca. 850 bp in length) were performed in CLC Genomics Workbench using the default base-calling quality and *de novo* assembly settings for Sanger sequence data. Phylogenetic analyses were performed on the Sanger-sequenced 16S sequences with maximum likelihood methods as described above.

The contributions of mutations and recombination to the genomic diversity in the concatenated core genome alignment, the number of recombination events (imports) per genome, and the positions of recombination hot spots were investigated using ClonalFrameML (26). This approach allows the assessment of homologous recombination within the core genome only (e.g., genes shared between all compared genomes) and does not assess nonhomologous gene acquisition (e.g., horizontal gene transfer) or gene loss events. Nucleotides unaffected by homologous recombination are referred to as unimported, and nucleotides subject to recombination are referred to as imported (26). ClonalFrameML provides the relative ratio of recombination to mutation (*R*/θ), the mean length of recombined DNA (δ), and the mean divergence of imported DNA (ν). These results were used to calculate the relative contribution of recombination versus mutation to the overall genomic diversity (*r*/*m*), using the formula *r*/*m* = (*R*/θ) × δ × ν. ClonalFrameML was performed in three separate runs, containing a core genome alignment that contained (i) all genomes, (ii) only the subseafloor genomes, and (iii) only the type strains. The resulting *r*/*m* values from these three groups (presented in Table 1) were then used to interpret the relative importance of mutations compared to recombination in the separate groups (e.g., type strains versus subseafloor strains). The *r*/*m* analysis provided by ClonalFrameML is capable of identifying recombination between closely related strains where the mean ν value can be as low as 0.031 (26). The mean ν value between our closely related subseafloor strains is slightly lower but comparable (ν = 0.026) (Table 1). The mean divergence of imported DNA that we describe is like those described in the original ClonalFrameML methods paper, suggesting that the *r*/*m* values that we calculated can detect recombination between the closely related subseafloor strains.

In addition to calculating sites and rates of recombination in the core genome, ClonalFrameML also estimates the ancestral sequences at internal nodes of the clonal genealogy and any missing base calls in the observed sequences. The reconstruction of ancestral sequence states is performed using maximum likelihood, and the ClonalFrame model can be thought of as a hidden Markov model (HMM) when the ancestral and descendant genomes for each branch of the clonal genealogy have been observed or reconstructed (26). The hidden state of the HMM records whether each nucleotide was subject to recombination or not on the branch connecting the two genomes. We acknowledge that drawing inference under the resulting ancestral recombination graph is a notoriously complex statistical problem (26). Instead, here, we use ClonalFrameML only to assess within-group recombination (e.g., between species within the genus *Thalassospira*), and thus, our analysis cannot assess the influence of external recombination (from species outside the genus *Thalassospira*).

The *dN*/*dS* ratio in the core genome alignment (global ω ratio) was estimated using HyPhy v2.2.4 (47) and applying the adaptive branch site random-effects likelihood (aBSREL) approach (48) to all branches in all subfamilies. The *dN*/*dS* calculation is based on an in-frame codon alignment of the core genome, together with the corresponding core genome phylogeny. As described previously by Smith et al. (48), aBSREL tests for each branch in the phylogeny (e.g., each genome in the core genome alignment) and whether a proportion of sites have evolved under positive selection and will infer the optimal number of ω classes (*dN*/*dS*) for each branch. aBSREL takes into consideration that different branches may feature evolutionary patterns with differing complexities and hence may be better modeled by more or fewer ω classes. To this end, aBSREL uses AICc (small-sample AIC [Akaike information criterion]) to infer the optimal number of ω for each branch in the core genome phylogeny. Tests for statistical significance of branches under positive selection (e.g., genomes with a significant *dN*/*dS* ratio) in aBSREL are based on likelihood ratio test (LRT) distributions, whereby a null model is defined in which no positive selection is allowed on a branch, and the LRT is used to determine whether the null model can be rejected (48). The aBSREL algorithm that calculates the global ω (*dN*/*dS*) ratio does not provide accurate results for positive selection when highly similar sequences are included in the in-frame codon alignment (48). Therefore, we calculated the *dN*/*dS* ratios from the in-frame codon core genome alignment with aBSREL 10 separate times, with each run containing one selected genome from each of the three subseafloor clades: (i) *T. xiamenensis* strain Neogene, (ii) *T. xiamenensis* strain Miocene, and (iii) “*Ca*. Thalassospira pliocenensis.” The results for each of these separate aBSREL runs are summarized in Table S2. By running aBSREL in this manner (multiple times using only one representative of highly similar subseafloor clades), statistically significant *dN*/*dS* values were obtained for each subseafloor genome (Table S2). The 10 different aBSREL run output files produced from the HyPhy program (.json files) were then visualized in HyPhy Vision (http://vision.hyphy.org/aBSREL), a Web graphical user interface that allows visualization of branch site selection, *P* values, and global ω (*dN*/*dS*) classes for each branch (genome) from the in-frame codon core genome alignment.

Low *dS* values may produce unreliable *dN*/*dS* ratios. aBSREL does not calculate separate *dN* or *dS* values. We investigated how the magnitude of *dS* in the subseafloor strains compared to that of the type strains using the FitMG94 workflow in HyPhy (https://github.com/veg/hyphy-analyses/tree/master/FitMG94) to fit the Muse-Gaut model of DNA sequence evolution (49) to the core genome alignment, which reports the number of synonymous and nonsynonymous substitutions per nucleotide site for each branch on the tree. The FitMG94 workflow also uses a corrected empirical estimator (CF3x4) that provides improved estimates of several parameters in the evolutionary model (50). The results of FitMG94 showed that there was no significant difference in the magnitude of *dS* in the type strains (0.02 to 0.36) compared to the subseafloor strains (0.001 to 0.07) (*P* = 0.59 by a two-sided *t* test).

### Pangenome analysis.

All subseafloor and type strain *Thalassospira* genomes were analyzed in Anvi’o v6.2 using the pangenome workflow (51). Briefly, each genome was converted into an Anvi’o contig database. Genes were functionally annotated using eggNOG v5.0 (52) with eggNOG-mapper (53) and imported back to each genome’s Anvi’o contig database. Genome storages were generated using anvi-gen-genomes-storages, and anvi-pan-genome was deployed with the parameters –min-bit 0.5, –mcl-inflation 10, and the flag –use-ncbi-blast. The Anvi’o pangenome database and a summary of gene clusters are stored in Figshare (https://figshare.com/s/06ba1287a00ab01a1ee).

### Identifying pseudogenes.

We estimated the number of pseudogenes within the genomes using two programs, Psi-Phi (54) and DFAST (55). Psi-Phi uses a conservative criterion considering a pseudogene only when it lost >20% of its original length and enhances pseudogene recognition among closely related strains both in annotated regions by identifying incorrectly annotated open reading frames (ORFs) and in intergenic regions by detecting new pseudogenes (54). Psi-Phi classifies pseudogenes as either identified pseudogenes or possible but potentially not pseudogenes. To be conservative, we considered only genes identified as pseudogenes from Psi-Phi and did not consider those flagged as potential pseudogenes. As a second check of the pseudogene content, we searched genomes for pseudogenes using DFAST (55). The estimated number of pseudogenes per genome was then taken as an average of the numbers detected using both methods (Psi-Phi and DFAST). On average, Psi-Phi identified a higher number of pseudogenes per genome (57 ± 10) than DFAST (32 ± 4), but the variation between methods for the same genome was consistent (average variation = 27; standard deviation of averages = 7). This minimal variation between individual genomes indicates that biases inherent to the pseudogene prediction methods affected the different genomes equally and thus allow for a pseudogene comparison between the genomes.

### Data availability.

Data are publicly available through NCBI BioProject accession number PRJNA473406. The raw genomic sequence reads are available in the SRA under BioSample accession numbers SAMN17168194, SAMN17168195, and SAMN17168196. The hybrid assemblies (contigs) for each of the subseafloor isolate genomes are available through the LMU Open Data database (https://doi.org/10.5282/ubm/data.233). The 16S data from the frozen core are available in the SRA under BioSample accession numbers SAMN10929403 to SAMN10929517. Figures and output files from the pangenomic analysis in Anvi’o are available at Figshare (https://doi.org/10.6084/m9.figshare.13372619). Additional data related to this paper may be requested from the authors.

## References

[1] Kallmeyer J, Pockalny R, Adhikari R, Smith DC, D’Hondt S. 2012. Global distribution of microbial abundance and biomass in subseafloor sediment. Proc Natl Acad Sci USA 109:16213–16216. doi:10.1073/pnas.1203849109. 22927371 PMC3479597

[2] Roy H, Kallmeyer J, Adhikari RR, Pockalny R, Jorgensen BB, D’Hondt S. 2012. Aerobic microbial respiration in 86-million-year-old deep-sea red clay. Science 336:922–925. doi:10.1126/science.1219424. 22605778

[3] D’Hondt S, Rutherford S, Spivack AJ. 2002. Metabolic activity of subsurface life in deep-sea sediments. Science 295:2067–2070. doi:10.1126/science.1064878. 11896277

[4] D’Hondt S, Spivack AJ, Pockalny R, Ferdelman TG, Fischer JP, Kallmeyer J, Abrams LJ, Smith DC, Graham D, Hasiuk F, Schrum H, Stancin AM. 2009. Subseafloor sedimentary life in the South Pacific Gyre. Proc Natl Acad Sci USA 106:11651–11656. doi:10.1073/pnas.0811793106. 19561304 PMC2702254

[5] D’Hondt S, Wang G, Spivack A. 2014. The underground economy (energetic constraints of subseafloor life), p 127–148. *In* Stein R, Blackman D, Inagaki F, Larsen H-C (ed), Earth and life processes discovered from subseafloor environment—a decade of science achieved by the Integrated Ocean Drilling Program (IODP). Elsevier, New York, NY.

[6] Hoehler TM, Jorgensen BB. 2013. Microbial life under extreme energy limitation. Nat Rev Microbiol 11:83–94. doi:10.1038/nrmicro2939. 23321532

[7] Orsi WD, Schink B, Buckel W, Martin WF. 2020. Physiological limits to life in anoxic subseafloor sediment. FEMS Microbiol Rev 44:219–231. doi:10.1093/femsre/fuaa004. 32065239 PMC7269680

[8] Cordero OX, Polz MF. 2014. Explaining microbial genomic diversity in light of evolutionary ecology. Nat Rev Microbiol 12:263–273. doi:10.1038/nrmicro3218. 24590245

[9] Biddle JF, Sylvan JB, Brazelton WJ, Tully BJ, Edwards K, Moyer CL, Heidelberg J, Nelson WC. 2012. Prospects for the study of evolution in the deep biosphere. Front Microbiol 2:285. doi:10.3389/fmicb.2011.00285. 22319515 PMC3265032

[10] Finkel SE. 2006. Long-term survival during stationary phase: evolution and the GASP phenotype. Nat Rev Microbiol 4:113–120. doi:10.1038/nrmicro1340. 16415927

[11] Wick LM, Weilenmann H, Egli T. 2002. The apparent clock-like evolution of Escherichia coli in glucose-limited chemostats is reproducible at large but not at small population sizes and can be explained with Monod kinetics. Microbiology (Reading) 148:2889–2902. doi:10.1099/00221287-148-9-2889. 12213934

[12] Starnawski P, Bataillon T, Ettema TJ, Jochum LM, Schreiber L, Chen X, Lever MA, Polz MF, Jorgensen BB, Schramm A, Kjeldsen KU. 2017. Microbial community assembly and evolution in subseafloor sediment. Proc Natl Acad Sci USA 114:2940–2945. doi:10.1073/pnas.1614190114. 28242677 PMC5358386

[13] Becraft ED, Lau Vetter MCY, Bezuidt OKI, Brown JM, Labonte JM, Kauneckaite-Griguole K, Salkauskaite R, Alzbutas G, Sackett JD, Kruger BR, Kadnikov V, van Heerden E, Moser D, Ravin N, Onstott T, Stepanauskas R. 6 April 2021. Evolutionary stasis of a deep subsurface microbial lineage. ISME J 10.1038/s41396-021-00965-3. PMC8443664 33824425

[14] Shapiro BJ, Friedman J, Cordero OX, Preheim SP, Timberlake SC, Szabo G, Polz M, Alm EJ. 2012. Population genomics of early events in the ecological differentiation of bacteria. Science 336:48–51. doi:10.1126/science.1218198. 22491847 PMC3337212

[15] Charlesworth B. 2009. Fundamental concepts in genetics: effective population size and patterns of molecular evolution and variation. Nat Rev Genet 10:195–205. doi:10.1038/nrg2526. 19204717

[16] Lomstein BA, Langerhuus AT, D’Hondt S, Jorgensen BB, Spivack AJ. 2012. Endospore abundance, microbial growth and necromass turnover in deep sub-seafloor sediment. Nature 484:101–104. doi:10.1038/nature10905. 22425999

[17] Vuillemin A, Wankel SD, Coskun OK, Magritsch T, Vargas S, Estes ER, Spivack AJ, Smith DC, Pockalny R, Murray RW, D’Hondt S, Orsi WD. 2019. Archaea dominate oxic subseafloor communities over multimillion-year time scales. Sci Adv 5:eaaw4108. doi:10.1126/sciadv.aaw4108. 31223656 PMC6584578

[18] Morono Y, Ito M, Hoshino T, Terada T, Hori T, Ikehara M, D’Hondt S, Inagaki F. 2020. Aerobic microbial life persists in oxic marine sediment as old as 101.5 million years. Nat Commun 11:3626. doi:10.1038/s41467-020-17330-1. 32724059 PMC7387439

[19] Li M, Yang S, Lai Q, Shao Z. 2017. Draft genome sequence of Thalassospira xiamenensis strain MCCC 1A03042^T^. Genome Announc 5:e01702-16. doi:10.1128/genomeA.01702-16. 28254975 PMC5334582

[20] Liu C, Wu Y, Li L, Ma Y, Shao Z. 2007. Thalassospira xiamenensis sp. nov. and Thalassospira profundimaris sp. nov. Int J Syst Evol Microbiol 57:316–320. doi:10.1099/ijs.0.64544-0. 17267971

[21] Hutz A, Schubert K, Overmann J. 2011. Thalassospira sp. isolated from the oligotrophic eastern Mediterranean Sea exhibits chemotaxis toward inorganic phosphate during starvation. Appl Environ Microbiol 77:4412–4421. doi:10.1128/AEM.00490-11. 21602377 PMC3127729

[22] Hungate BA, Mau RL, Schwartz E, Caporaso JG, Dijkstra P, van Gestel N, Koch BJ, Liu CM, McHugh TA, Marks JC, Morrissey EM, Price LB. 2015. Quantitative microbial ecology through stable isotope probing. Appl Environ Microbiol 81:7570–7581. doi:10.1128/AEM.02280-15. 26296731 PMC4592864

[23] Rodriguez-R LM, Konstantinidis K. 2014. Bypassing cultivation to identify bacterial species. Microbe 9:111–118. doi:10.1128/microbe.9.111.1.

[24] Vos M, Didelot X. 2009. A comparison of homologous recombination rates in bacteria and archaea. ISME J 3:199–208. doi:10.1038/ismej.2008.93. 18830278

[25] Lever MA, Rogers KL, Lloyd KG, Overmann J, Schink B, Thauer RK, Hoehler TM, Jorgensen BB. 2015. Life under extreme energy limitation: a synthesis of laboratory- and field-based investigations. FEMS Microbiol Rev 39:688–728. doi:10.1093/femsre/fuv020. 25994609

[26] Didelot X, Wilson DJ. 2015. ClonalFrameML: efficient inference of recombination in whole bacterial genomes. PLoS Comput Biol 11:e1004041. doi:10.1371/journal.pcbi.1004041. 25675341 PMC4326465

[27] Koskiniemi S, Sun S, Berg OG, Andersson DI. 2012. Selection-driven gene loss in bacteria. PLoS Genet 8:e1002787. doi:10.1371/journal.pgen.1002787. 22761588 PMC3386194

[28] Drake JW, Charlesworth B, Charlesworth D, Crow JF. 1998. Rates of spontaneous mutation. Genetics 148:1667–1686. doi:10.1093/genetics/148.4.1667. 9560386 PMC1460098

[29] McCutcheon JP, Moran NA. 2011. Extreme genome reduction in symbiotic bacteria. Nat Rev Microbiol 10:13–26. doi:10.1038/nrmicro2670. 22064560

[30] Muller HJ. 1964. The relation of recombination to mutational advance. Mutat Res 106:2–9. doi:10.1016/0027-5107(64)90047-8. 14195748

[31] Kuo CH, Moran NA, Ochman H. 2009. The consequences of genetic drift for bacterial genome complexity. Genome Res 19:1450–1454. doi:10.1101/gr.091785.109. 19502381 PMC2720180

[32] Lloyd KG, Steen AD, Ladau J, Yin J, Crosby L. 2018. Phylogenetically novel uncultured microbial cells dominate earth microbiomes. mSystems 3:e00055-18. doi:10.1128/mSystems.00055-18. 30273414 PMC6156271

[33] D’Hondt S, Inagaki F, Zarikian CA, Abrams LJ, Dubois N, Engelhardt T, Evans H, Ferdelman T, Gribsholt B, Harris RN, Hoppie BW, Hyun J-H, Kallmeyer J, Kim J, Lynch JE, McKinley CC, Mitsunobu S, Morono Y, Murray RW, Pockalny R, Sauvage J, Shimono T, Shiraishi F, Smith DC, Smith-Duque CE, Spivack AJ, Steinsbu BO, Suzuki Y, Szpak M, Toffin L, Uramoto G, Yamaguchi YT, Zhang G, Zhang X-H, Ziebis W. 2015. Presence of oxygen and aerobic communities from sea floor to basement in deep-sea sediments. Nat Geosci 8:299–304. doi:10.1038/ngeo2387.

[34] Teal LR, Bulling MT, Parker ER, Solan M. 2008. Global patterns of bioturbation intensity and mixed depth of marine soft sediments. Aquat Biol 2:207–218. doi:10.3354/ab00052.

[35] Pichler M, Coskun OK, Ortega-Arbulu AS, Conci N, Worheide G, Vargas S, Orsi WD. 2018. A 16S rRNA gene sequencing and analysis protocol for the Illumina MiniSeq platform. Microbiologyopen 7:e00611. doi:10.1002/mbo3.611. 29575567 PMC6291791

[36] Ortega-Arbulu AS, Pichler M, Vuillemin A, Orsi WD. 2019. Effects of organic matter and low oxygen on the mycobenthos in a coastal lagoon. Environ Microbiol 21:374–388. doi:10.1111/1462-2920.14469. 30411473 PMC7379666

[37] Coskun OK, Ozen V, Wankel SD, Orsi WD. 2019. Quantifying population-specific growth in benthic bacterial communities under low oxygen using H2(18)O. ISME J 13:1546–1559. doi:10.1038/s41396-019-0373-4. 30783213 PMC6776007

[38] Mashimo K, Nagata Y, Kawata M, Iwasaki H, Yamamoto K. 2004. Role of the RuvAB protein in avoiding spontaneous formation of deletion mutations in the Escherichia coli K-12 endogenous tonB gene. Biochem Biophys Res Commun 323:197–203. doi:10.1016/j.bbrc.2004.08.078. 15351721

[39] Wick RR, Judd LM, Gorrie CL, Holt KE. 2017. Unicycler: resolving bacterial genome assemblies from short and long sequencing reads. PLoS Comput Biol 13:e1005595. doi:10.1371/journal.pcbi.1005595. 28594827 PMC5481147

[40] Parks DH, Imelfort M, Skennerton CT, Hugenholtz P, Tyson GW. 2015. CheckM: assessing the quality of microbial genomes recovered from isolates, single cells, and metagenomes. Genome Res 25:1043–1055. doi:10.1101/gr.186072.114. 25977477 PMC4484387

[41] Overbeek R, Olson R, Pusch GD, Olsen GJ, Davis JJ, Disz T, Edwards RA, Gerdes S, Parrello B, Shukla M, Vonstein V, Wattam AR, Xia F, Stevens R. 2014. The SEED and the Rapid Annotation of Microbial Genomes Using Subsystems Technology (RAST). Nucleic Acids Res 42:D206–D214. doi:10.1093/nar/gkt1226. 24293654 PMC3965101

[42] Edgar RC. 2004. MUSCLE: a multiple sequence alignment method with reduced time and space complexity. BMC Bioinformatics 5:113. doi:10.1186/1471-2105-5-113. 15318951 PMC517706

[43] Guindon S, Lethiec F, Duroux P, Gascuel O. 2005. PHYML Online—a Web server for fast maximum likelihood-based phylogenetic inference. Nucleic Acids Res 33:W557–W559. doi:10.1093/nar/gki352. 15980534 PMC1160113

[44] Gouy M, Guindon S, Gascuel O. 2010. SeaView version 4: a multiplatform graphical user interface for sequence alignment and phylogenetic tree building. Mol Biol Evol 27:221–224. doi:10.1093/molbev/msp259. 19854763

[45] Lane DJ. 1991. 16S/23S rRNA sequencing, p 115–175. *In* Stackebrandt E, Goodfellow M (ed), Nucleic acid techniques in bacterial systematics. John Wiley & Sons, New York, NY.

[46] Turner S, Pryer KM, Miao VPW, Palmer JD. 1999. Investigating deep phylogenetic relationships among cyanobacteria and plastids by small subunit rRNA sequence analysis. J Eukaryot Microbiol 46:327–338. doi:10.1111/j.1550-7408.1999.tb04612.x. 10461381

[47] Kosakovsky Pond SL, Frost SD, Muse SV. 2005. HyPhy: hypothesis testing using phylogenies. Bioinformatics 21:676–679. doi:10.1093/bioinformatics/bti079. 15509596

[48] Smith MD, Wertheim JO, Weaver S, Murrell B, Scheffler K, Kosakovsky Pond SL. 2015. Less is more: an adaptive branch-site random effects model for efficient detection of episodic diversifying selection. Mol Biol Evol 32:1342–1353. doi:10.1093/molbev/msv022. 25697341 PMC4408413

[49] Muse SV, Gaut BS. 1994. A likelihood approach for comparing synonymous and nonsynonymous nucleotide substitution rates, with application to the chloroplast genome. Mol Biol Evol 11:715–724. doi:10.1093/oxfordjournals.molbev.a040152. 7968485

[50] Kosakovsky Pond S, Delport W, Muse SV, Scheffler K. 2010. Correcting the bias of empirical frequency parameter estimators in codon models. PLoS One 5:e11230. doi:10.1371/journal.pone.0011230. 20689581 PMC2912764

[51] Eren AM, Esen OC, Quince C, Vineis JH, Morrison HG, Sogin ML, Delmont TO. 2015. Anvi’o: an advanced analysis and visualization platform for ‘omics data. PeerJ 3:e1319. doi:10.7717/peerj.1319. 26500826 PMC4614810

[52] Huerta-Cepas J, Szklarczyk D, Heller D, Hernandez-Plaza A, Forslund SK, Cook H, Mende DR, Letunic I, Rattei T, Jensen LJ, von Mering C, Bork P. 2019. eggNOG 5.0: a hierarchical, functionally and phylogenetically annotated orthology resource based on 5090 organisms and 2502 viruses. Nucleic Acids Res 47:D309–D314. doi:10.1093/nar/gky1085. 30418610 PMC6324079

[53] Huerta-Cepas J, Forslund K, Coelho LP, Szklarczyk D, Jensen LJ, von Mering C, Bork P. 2017. Fast genome-wide functional annotation through orthology assignment by eggNOG-Mapper. Mol Biol Evol 34:2115–2122. doi:10.1093/molbev/msx148. 28460117 PMC5850834

[54] Lerat E, Ochman H. 2004. Psi-Phi: exploring the outer limits of bacterial pseudogenes. Genome Res 14:2273–2278. doi:10.1101/gr.2925604. 15479949 PMC525686

[55] Tanizawa Y, Fujisawa T, Nakamura Y. 2018. DFAST: a flexible prokaryotic genome annotation pipeline for faster genome publication. Bioinformatics 34:1037–1039. doi:10.1093/bioinformatics/btx713. 29106469 PMC5860143

