# Supplementary figures and images for: Genome Evolution in Bacteria Isolated from Million-Year-Old Subseafloor Sediment
Source: mBio. 2021 Aug 17;12(4):e01150-21. doi: 10.1128/mBio.01150-21 (PMC8406185; doi:10.1128/mBio.01150-21)

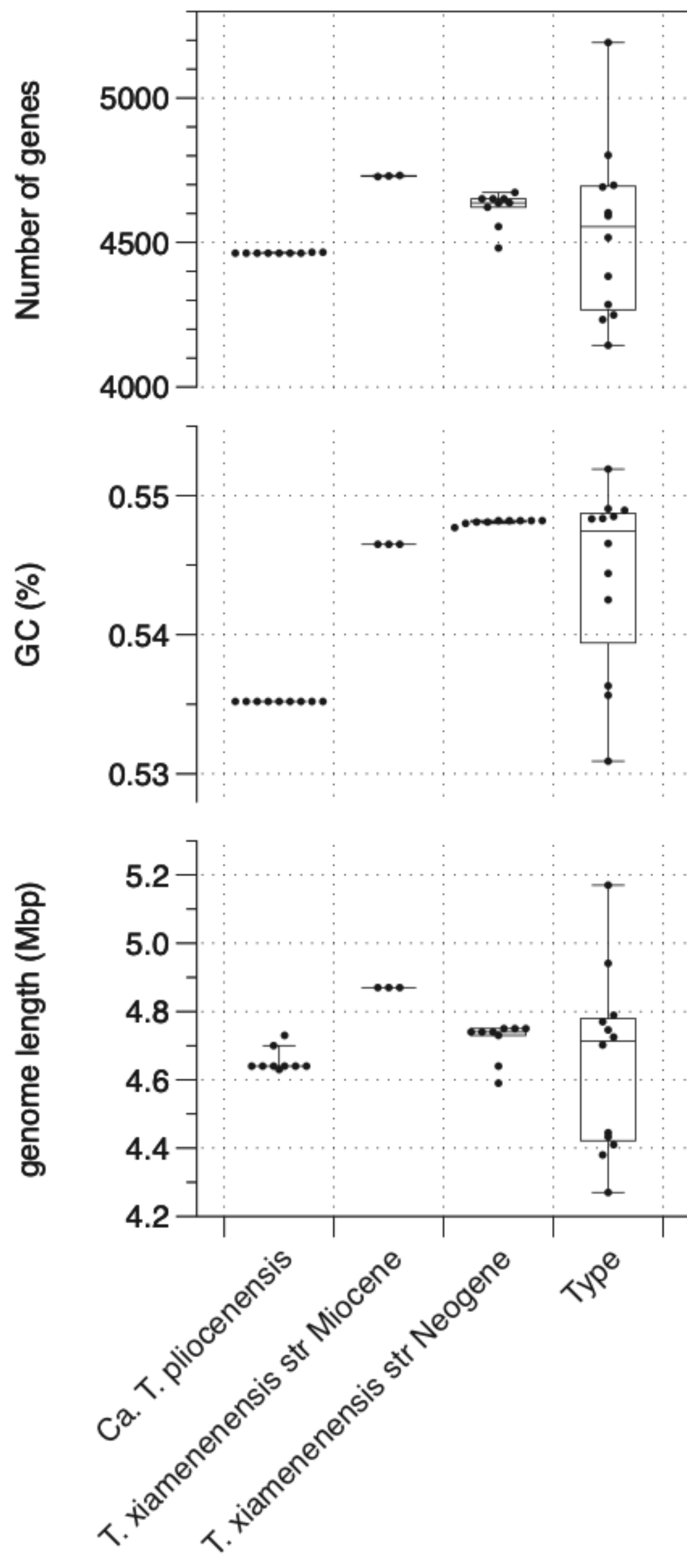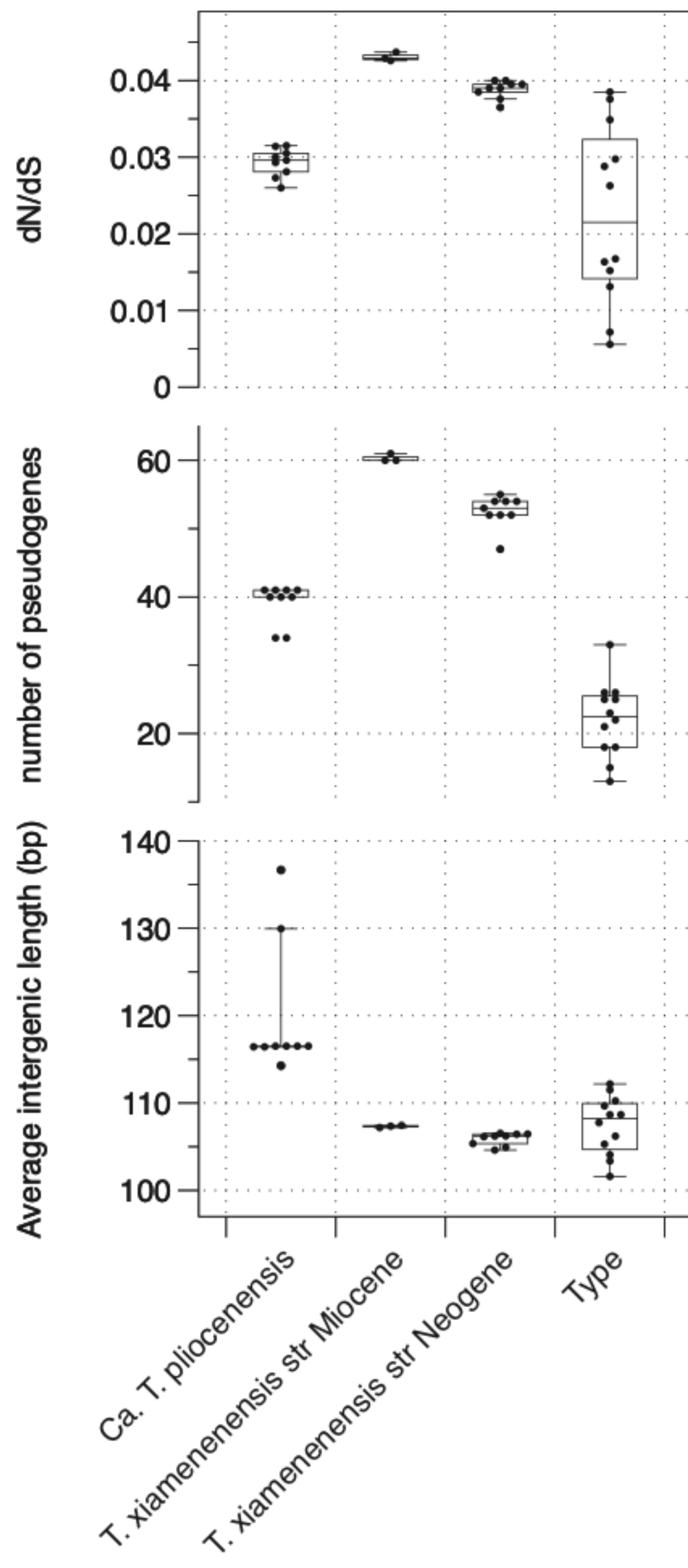

Supplement: FIG S1 [file mbio.01150-21-sf001.pdf]

Number of nucleotide differences

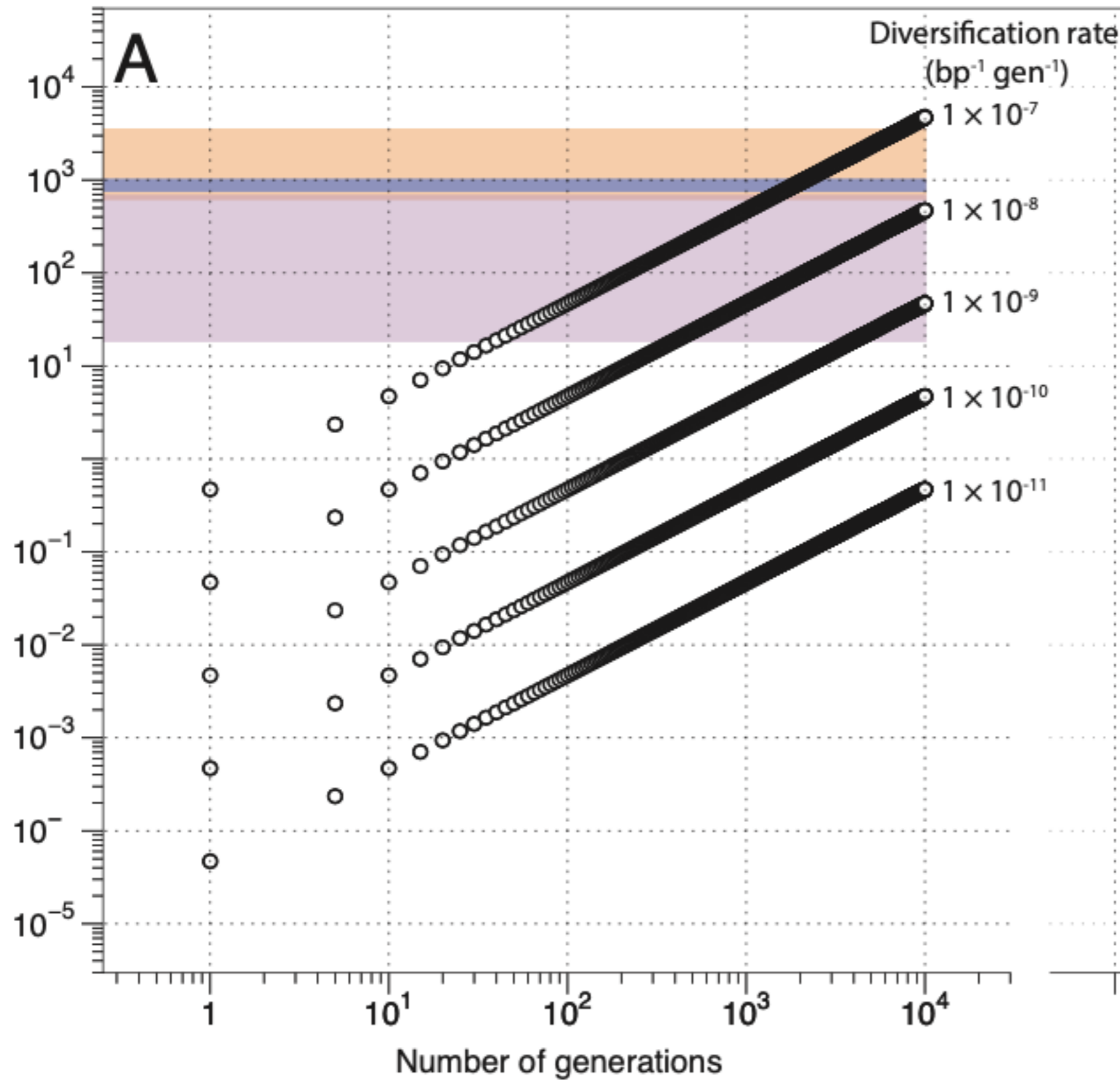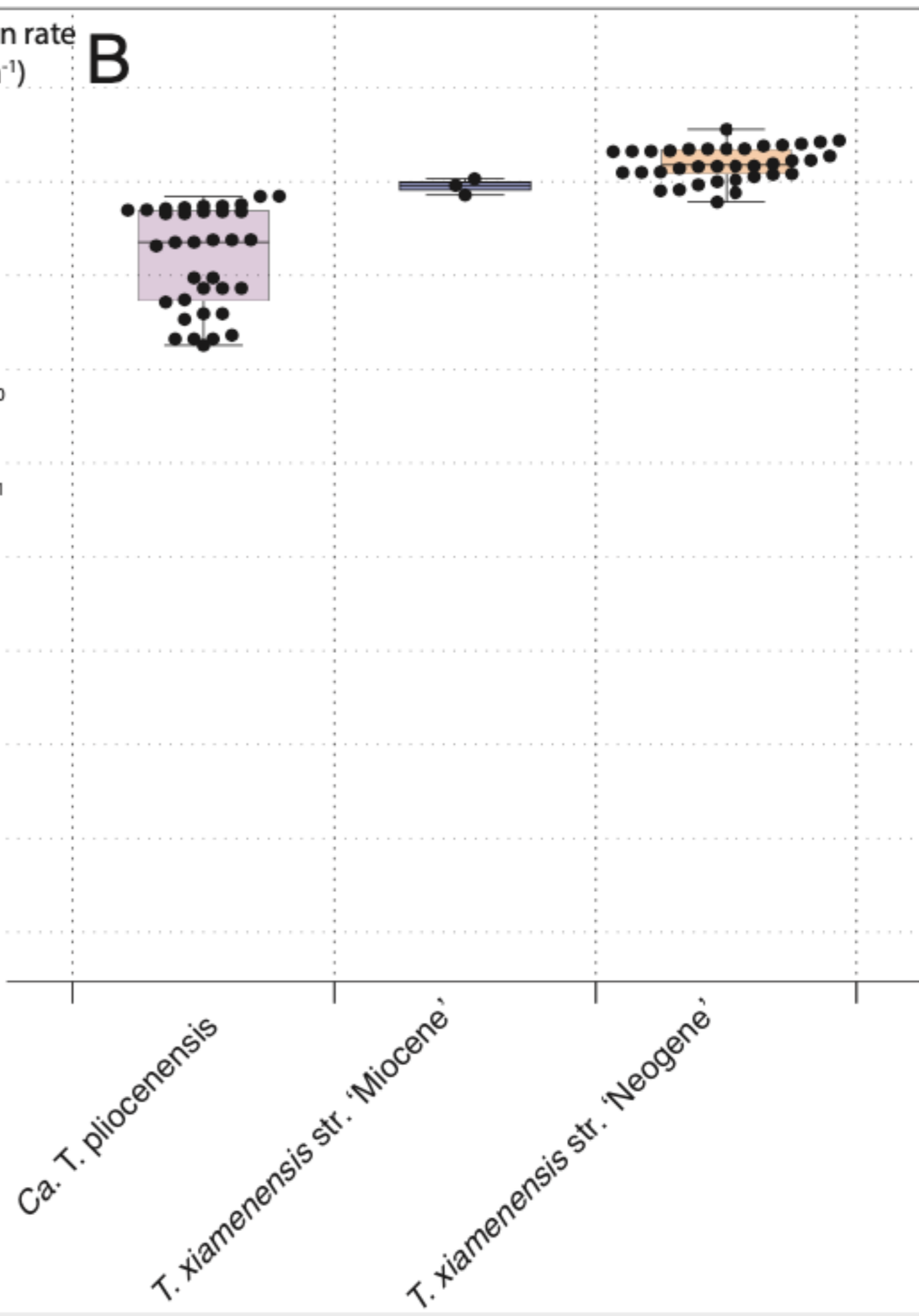

Supplement: FIG S4 [file mbio.01150-21-sf004.pdf]

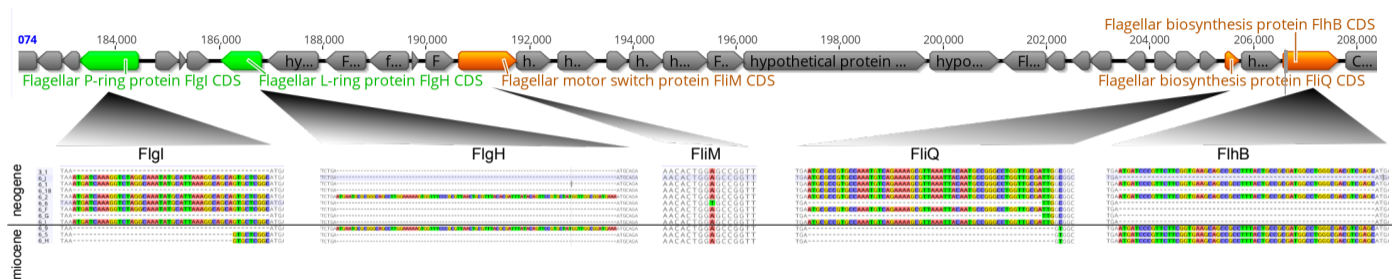

Supplement: FIG S5 [file mbio.01150-21-sf005.pdf]
