# Supplementary material for: Genome Evolution in Bacteria Isolated from Million-Year-Old Subseafloor Sediment
Source: mBio. 2021 Aug 17;12(4):e01150-21. doi: 10.1128/mBio.01150-21 (PMC8406185; doi:10.1128/mBio.01150-21)
Supplement: FIG S2 [file mbio.01150-21-sf002.pdf]

		'*Ca*. T. pliocienensis'									*T. xiamenensis* str. 'Miocene'			*T. xiamenensis* str. 'Neogene'								
		3_3	3_4	3_6	3_7	3_F	3_G	3_H	3_I	3_J	6_9	6_5	6_H	3_1	6_1	6_2	6_6	6_G	6_18	6_F	6_I	6_J
'*Ca*. T. pliocienensis'	3_3		225	206	240	226	239	239	695	698	403,756	403,358	403,350	403,126	402,924	403,920	403,178	402,966	403,318	403,762	403,239	403,517
	3_4	225		21	55	73	94	94	550	569	403,771	403,374	403,365	403,140	402,939	403,935	403,193	402,981	403,333	403,776	403,253	403,532
	3_6	206	21		34	52	73	73	529	548	403,764	403,367	403,358	403,133	402,932	403,928	403,186	402,974	403,326	403,769	403,246	403,525
	3_7	240	55	34		18	39	39	495	514	403,768	403,371	403,362	403,137	402,936	403,932	403,190	402,978	403,330	403,773	403,250	403,529
	3_F	226	73	52	18		21	21	477	500	403,760	403,363	403,354	403,129	402,928	403,924	403,182	402,970	403,322	403,765	403,242	403,521
	3_G	239	94	73	39	21		0	456	479	403,758	403,361	403,352	403,126	402,925	403,921	403,179	402,967	403,319	403,762	403,239	403,518
	3_H	239	94	73	39	21	0		456	479	403,758	403,361	403,352	403,126	402,925	403,921	403,179	402,967	403,319	403,762	403,239	403,518
	3_I	695	550	529	495	477	456	456		23	403,864	403,467	403,458	403,230	403,029	404,025	403,283	403,071	403,423	403,866	403,343	403,622
	3_J	698	569	548	514	500	479	479	23		403,863	403,466	403,457	403,229	403,028	404,024	403,282	403,070	403,422	403,865	403,342	403,621
T. xiamenensis str. 'Miocene'	6_9	403,756	403,771	403,764	403,768	403,760	403,758	403,758	403,864	403,863		1,071	909	29,065	29,754	30,305	29,775	29,461	29,826	30,652	29,952	29,949
	6_5	403,358	403,374	403,367	403,371	403,363	403,361	403,361	403,467	403,466	1,071		720	29,269	29,485	30,167	29,686	29,156	29,425	30,749	30,145	29,710
	6_H	403,350	403,365	403,358	403,362	403,354	403,352	403,352	403,458	403,457	909	720		28,731	29,354	30,138	29,555	29,127	29,294	30,102	29,498	29,579
T. xiamenensis str. 'Neogene'	3_1	403,126	403,140	403,133	403,137	403,129	403,126	403,126	403,230	403,229	29,065	29,269	28,731		1,465	2,239	1,547	1,128	1,380	2,084	1,461	1,683
	6_1	402,924	402,939	402,932	402,936	402,928	402,925	402,925	403,029	403,028	29,754	29,485	29,354	1,465		2,232	760	1,213	1,245	2,132	924	1,038
	6_2	403,920	403,935	403,928	403,932	403,924	403,921	403,921	404,025	404,024	30,305	30,167	30,138	2,239	2,232		2,452	2,101	2,103	3,602	2,646	2,394
	6_6	403,178	403,193	403,186	403,190	403,182	403,179	403,179	403,283	403,282	29,775	29,686	29,555	1,547	760	2,452		797	997	2,214	820	1,252
	6_G	402,966	402,981	402,974	402,978	402,970	402,967	402,967	403,071	403,070	29,461	29,156	29,127	1,128	1,213	2,101	797		606	2,227	1,190	1,671
	6_18	403,318	403,333	403,326	403,330	403,322	403,319	403,319	403,423	403,422	29,826	29,425	29,294	1,380	1,245	2,103	997	606		2,521	1,269	1,466
	6_F	403,762	403,776	403,769	403,773	403,765	403,762	403,762	403,866	403,865	30,652	30,749	30,102	2,084	2,132	3,602	2,214	2,227	2,521		1,875	2,738
	6_I	403,239	403,253	403,246	403,250	403,242	403,239	403,239	403,343	403,342	29,952	30,145	29,498	1,461	924	2,646	820	1,190	1,269	1,875		1,448
	6_J	403,517	403,532	403,525	403,529	403,521	403,518	403,518	403,622	403,621	29,949	29,710	29,579	1,683	1,038	2,394	1,252	1,671	1,466	2,738	1,448	
