# Supplementary material for: Genome Evolution in Bacteria Isolated from Million-Year-Old Subseafloor Sediment
Source: mBio. 2021 Aug 17;12(4):e01150-21. doi: 10.1128/mBio.01150-21 (PMC8406185; doi:10.1128/mBio.01150-21)
Supplement: FIG S3 [file mbio.01150-21-sf003.pdf]

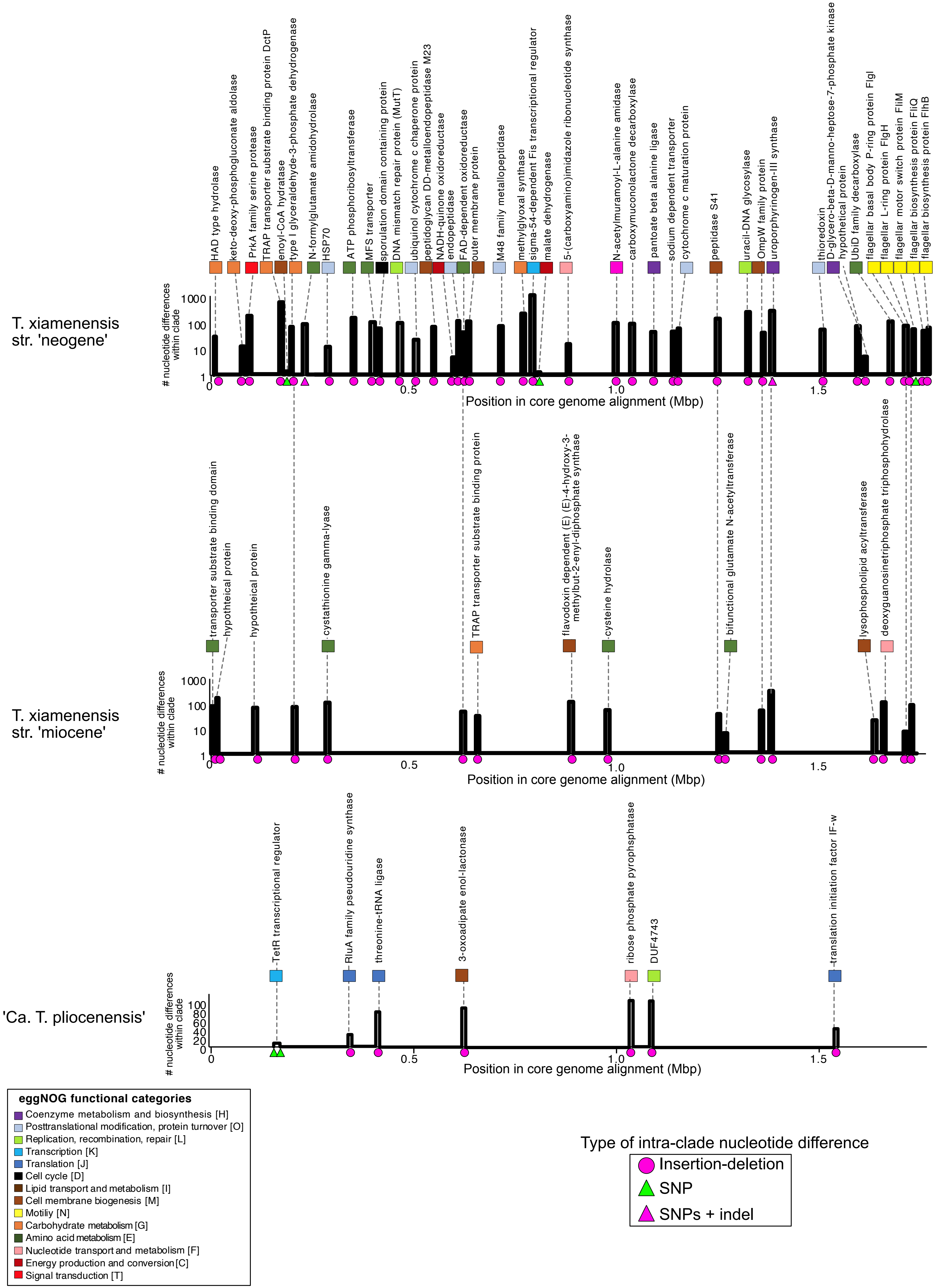

HAD type hydrolase
keto-deoxy-phosphogluconate aldolase
PrkA family serine protease
TRAP transporter substrate binding protein DctP
enoyl-CoA hydratase
type I glyceraldehyde-3-phosphate dehydrogenase
N-formylglutamate amidohydrolase
HSP70
ATP phosphoribosyltransferase
MFS transporter
sporulation domain containing protein
DNA mismatch repair protein (MutT)
ubiquinol cytochrome c chaperone protein
peptidoglycan DD-metalloendopeptidase M23
NADH-quinone oxidoreductase
endopeptidase
FAD-dependent oxidoreductase
outer membrane protein
M48 family metallopeptidase
methylglyoxal synthase
sigma-54-dependent Fis transcriptional regulator
malate dehydrogenase
5-(carboxyamino)imidazole ribonucleotide synthase
N-acetylmuramoyl-L-alanine amidase
carboxymuconolactone decarboxylase
pantoate beta alanine ligase
sodium dependent transporter
cytochrome c maturation protein
peptidase S41
uracil-DNA glycosylase
OmpW family protein
uroporphyrinogen-III synthase
thioredoxin
D-glycero-beta-D-manno-heptose-7-phosphate kinase
hypothetical protein
UbiD family decarboxylase
flagellar basal body P-ring protein FlgI
flagellar L-ring protein FlgH
flagellar motor switch protein FliM
flagellar biosynthesis protein FliQ
flagellar biosynthesis protein FlhB
T. xiamenensis str. 'neogene'
nucleotide differences within clade
1000
100
10
1
0
0.5
1.0
1.5
Position in core genome alignment (Mbp)
transporter substrate binding domain
hypothteical protein
hypothteical protein
cystathionine gamma-lyase
TRAP transporter substrate binding protein
flavodoxin dependent (E) (E)-4-hydroxy-3-methylbut-2-enyl-diphosphate synthase
cysteine hydrolase
bifunctional glutamate N-acetyltransferase
lysophospholipid acyltransferase
deoxyguanosinetriphosphate triphosphohydrolase
T. xiamenensis str. 'miocene'
nucleotide differences within clade
1000
100
10
1
0
0.5
1.0
1.5
Position in core genome alignment (Mbp)
TetR transcriptional regulator
RluA family pseudouridine synthase
threonine-tRNA ligase
3-oxoadipate enol-lactonase
ribose phosphate pyrophsphatase
DUF4743
translation initiation factor IF-w
'Ca. T. pliocenensis'
nucleotide differences within clade
100
80
60
40
20
0
0
0.5
1.0
1.5
Position in core genome alignment (Mbp)
eggNOG functional categories
Coenzyme metabolism and biosynthesis [H]
Posttranslational modification, protein turnover [O]
Replication, recombination, repair [L]
Transcription [K]
Translation [J]
Cell cycle [D]
Lipid transport and metabolism [I]
Cell membrane biogenesis [M]
Motiliy [N]
Carbohydrate metabolism [G]
Amino acid metabolism [E]
Nucleotide transport and metabolism [F]
Energy production and conversion [C]
Signal transduction [T]
Type of intra-clade nucleotide difference
Insertion-deletion
SNP
SNPs + indel
